# Dietary supplementation with 1‐kestose induces altered locomotor activity and increased striatal dopamine levels with a change in gut microbiota in male mice

**DOI:** 10.14814/phy2.15882

**Published:** 2023-12-06

**Authors:** Altanzul Altaisaikhan, Kazufumi Yoshihara, Tomokazu Hata, Noriyuki Miyata, Yasunari Asano, Takafumi Suematsu, Yoshihiro Kadota, Nobuyuki Sudo

**Affiliations:** ^1^ Department of Psychosomatic Medicine, Graduate School of Medical Sciences Kyushu University Fukuoka Japan; ^2^ Research and Development Center, B Food Science Co., Ltd. Chita Japan

**Keywords:** 1‐kestose, gut microbiota, locomotor activity, microbial diversity, striatal dopamine

## Abstract

1‐Kestose (KES), a dietary fiber and prebiotic carbohydrate, benefits various physiological functions. This study aimed to examine whether diets supplemented with KES over three consecutive generations could significantly affect some host physiological aspects, including behavioral phenotypes and gut microbial ecology. Mice that received KES‐supplemented diets for three generations demonstrated increased activity compared with those fed diets lacking KES. Furthermore, the KES group showed increased striatal dopamine (DA) and serotonin (5‐HT) levels. The observed increase in DA levels within the striatum was positively correlated with locomotor activity in the KES group but not in the control (CON) group. The α‐diversities were significantly lower in the KES group compared to the CON group. The three‐dimensional principal coordinate analysis revealed a substantial distinction between the KES and CON groups across each generation. At the genus level, most gut microbiota genera exhibited lower abundances in the KES group than in the CON group, except for *Bifidobacteria* and *Akkermansia*. Spearman's rank‐order analysis indicated significant negative correlations between the striatal DA levels and α‐diversity values. These findings suggest that prolonged supplementation with KES may stimulate increased locomotor activity along with elevated striatal DA levels, which are potentially associated with KES‐induced alterations in the gut microbiota.

## 1 INTRODUCTION

Different types of diets, such as Western and plant‐based diets, can have distinct effects on the gut microbiota. For example, a Western diet, which typically includes high levels of saturated fats, refined sugars, and processed foods (Martinez‐Gonzalez & Bes‐Rastrollo, 2014; Mozaffarian et al., 2011; Popkin et al., 2012), has been associated with a decrease in microbial diversity and an increase in certain bacteria (Statovci et al., 2017). However, microbial diversity is contentious as a measure of host health as well as of developmental cognitive function (Carlson et al., 2018). In contrast, a plant‐based diet rich in fiber, fruits, vegetables, and whole grains promotes a more diverse gut microbiota (Tomova et al., 2019).

1‐Kestose (KES) is a fructo‐oligosaccharide, a type of dietary fiber, and a prebiotic carbohydrate found in various plant sources (Suzuki et al., 2006). After consumption, KES moves through the gastrointestinal tract and reaches the large intestine, where it undergoes fermentation by gut bacteria. This fermentation process generates short‐chain fatty acids, which provide energy to the cells lining the colon and help regulate various host functions (Cummings, 1981). Supplementation with KES increases the abundance of some bacteria, such as *Bifidobacterium* and *Lactobacillus* (Tochio et al., 2016). Additionally, KES supplementation suppresses the development of diabetes in type 2 diabetes‐prone rats (Watanabe et al., 2020) and improves insulin sensitivity in overweight adults (Watanabe, Tochio, et al., 2021).

More than 1000 species of microbes, referred to as the gut microbiota, are present in the human gut (Qin et al., 2010). Moreover, Sender et al. described that the number of bacteria in the body is actually of the same order as the number of human cells and that their total mass is approximately 0.2 kg (Sender et al., 2016). Several recent studies have indicated that gut microbes can play a role in regulating body weight (Dabke et al., 2019) and the pathophysiology of the brain (Sampson et al., 2016). The gut microbiome influences the stress response of the central nervous system (Sudo et al., 2004) and other behaviors (Clarke et al., 2013; Heijtz et al., 2011; Neufeld et al., 2011; Nishino et al., 2013). This concept, now called the microbiota–gut–brain axis (Collins et al., 2012; Cryan & Dinan, 2012; Forsythe et al., 2010), is a model indicating refined cross‐talks between the microbiota, gut, and brain. In addition, Li et al. demonstrated the role of diet in driving microbiota changes in regulating host behavior (Li et al., 2009).

Therefore, it is intriguing to speculate that dietary components like KES can influence the host's behavior by modulating the microbiota‐gut‐brain axis. However, little is known about the potential effects of long‐term dietary consumption of KES across multiple generations on behavioral phenotypes. Hence, the present study aimed to investigate whether diets supplemented with KES over three consecutive generations could significantly affect physiological aspects, including body weight gain and behavioral phenotypes, along with their effects on gut microbial ecology.

## 2 METHODS

### 2.1 Animals

Four‐week‐old male and female BALB/c mice were obtained from KBT Oriental Co. Ltd. (Saga, Japan). The mice were housed individually and maintained under previously described conditions (Asano et al., 2012; Kimura‐Todani et al., 2020). The cages were made of plastic with wiretops. Animal experiments were approved by the Ethics Committee on Animal Experiments of the Graduate School of Medical Sciences, Kyushu University (A20‐175‐0) and were conducted in compliance with the Guidelines for Animal Experiments of the Graduate School of Medical Sciences, Kyushu University, as well as the Law (No. 105) and Notification (No. 6) of the Japanese Government.

### 2.2 Study protocol

Mice in the first generation (G1) received an isocaloric diet supplemented with 5% KES (KES, B Food Science Co., Ltd., Chita, Japan) or no supplement (CON) when they reached 5 weeks of age. The composition of the CON diet was based on the AIN‐93G diet (Reeves et al., 1993), and an equivalent quantity of KES was used to replace sucrose in the KES diet. Feed was provided as pelleted food. The mice were divided into two distinct groups. One group was used for behavioral experiments and sample collection, as detailed below, and the other group was designated for breeding purposes. Breeding was performed according to the following protocols. Briefly, at 8 weeks of age, male and female mice were placed together in a cage for mating. Male/female pairs from the CON and KES groups were maintained on CON and KES diets, respectively. Approximately 3 weeks after mating, 5–10 offspring were born per mother, collectively referred to as the second generation (G2). The G2 offspring, divided into the KES and CON groups, were subjected to the same treatment as the G1 mice. Subsequently, the offspring born to G2 mice, referred to as the third generation (G3), underwent the same behavioral evaluation protocol as G1 and G2 mice. G2 and G3 mice were similarly housed individually after weaning, and then fed CON or KES diets immediately after weaning. Only male pups were carried forward, labeled as G2 and G3, and used in the experiments. Female pups were also maintained for mating in a separate cage.

Body weight and food intake were measured twice weekly throughout the observation period. Behavioral experiments, namely the open‐field (OF), elevated plus‐maze (EPM), and forced swimming (FS) tests, were conducted on all mice between 8 and 10 weeks of age. Subsequently, blood samples were collected via cardiac puncture under anesthesia (using 0.3 mg/kg medetomidine, 4 mg/kg midazolam, and 5 mg/kg butorphanol). One or two days after the last behavioral test, the mice were euthanized by cervical dislocation, and the brain and fecal samples were collected and promptly frozen on dry ice. These samples were then stored at −80°C until further analyses.

### 2.3 Behavioral analyses

Behavioral assessments of the mice were conducted using OF, EPM, and FS tests, as described previously (Hata et al., 2019; Kimura‐Todani et al., 2020; Suemaru et al., 2006). These tests were administered under low illumination (<50 lx) between 9:00 am and 5:00 pm, following the initiation of dietary manipulation in the following sequence: OF, EPM, and FS tests. The tests were performed alternately among the two groups to minimize the impact of time‐related variations.

The OF test was used to evaluate anxiety levels and locomotor activity. Briefly, the mice were placed individually in the center of an OF box (45 × 45 × 45 cm, L × B × H) divided equally into 16 sub‐squares (4 × 4) (Kimura‐Todani et al., 2020; Nishino et al., 2013). Behaviors were recorded and quantified using a SMART3.0 computer system (Panlab Harvard Apparatus, USA). The total distance traveled in 20 min and the time spent in the 12 peripheral sub‐squares over 20 min were calculated as representative parameters of spontaneous locomotor activity and anxiety‐like behavior, respectively. Prior to testing each animal, the entire OF arena was cleaned with a 70% ethyl alcohol solution and dried with paper towels (Gunn et al., 2011) before the next mouse.

The EPM test was performed as previously described (Kimura‐Todani et al., 2020; Walf & Frye, 2007). The apparatus consisted of two open (30 × 5 cm) and two closed (30 × 5 × 15 cm) arms extending from a central platform (5 × 5 cm) and the test was administered in a quiet room illuminated with dim light. The number of entries into the open or closed arms and the time spent in the open or closed arms during the test period were recorded. The time spent in open arms was used as a parameter of anxiety‐like behavior. The total distance traveled was used as a parameter of locomotor activity. An entry was defined as the center of mass of the mouse entering the arm. The behavior of the tested animal was recorded for 5 min using a fully automated system. After each trial, the maze was cleaned with 70% ethyl alcohol solution and dried with paper towels (Gunn et al., 2011) before the next mouse.

The FS test was performed according to the procedures described elsewhere (Suemaru et al., 2006). Briefly, mice were placed in a cylindrical container filled with water (temperature 23–24°C, water depth 15 cm). The duration of immobility during a 6‐min session was recorded and used as an indicator of depression‐like behavior. A video camera‐based computer tracking system (SMART3.0, Panlab Harvard Apparatus, USA) was used to record the activities during each test. All behavioral tests were evaluated by researchers who were blinded to experimental conditions.

### 2.4 Measurement of monoamine levels in the brain

Monoamines and their metabolites were quantified as previously described (Hata et al., 2017; Nishino et al., 2013; Watanabe, Mikami, et al., 2021). All mice were euthanized by cervical dislocation at 8 weeks of age, and the blood, brain, and fecal material were collected. The excised brains were dissected into the medial prefrontal cortex, striatum, hippocampus, and brain stem. Each sample was prepared as previously described (Nishino et al., 2013; Zhang et al., 2022). Briefly, the samples were homogenized in 0.2 M perchloric acid containing 100 mM disodium EDTA. The homogenate was incubated for 30 min for deproteinization and then centrifuged at 20,000 × *g* for 10 min at 4°C. The pH of the supernatant was adjusted to approximately 3.0 by adding 1 M sodium acetate, and the resultant supernatant was filtered through a 0.22 μm filter (Merck Millipore Ltd., Ireland). The resultant 30 μL of filtrate was injected into an HPLC system (Eicom, Kyoto, Japan) equipped with a 150 × 3.0 mm octadecyl silane column (SC‐5ODS, Eicom) and an electrochemical detector (ECD‐300, Eicom, Kyoto, Japan) set at an applied potential of +0.75 V versus an Ag/Ag Cl reference analytical electrode. The levels of monoamines, dopamine (DA) metabolites (dihydroxyphenylacetic acid [DOPAC] and HVA), NE metabolites (MHPG), and 5‐HT metabolite (5‐hydroxyindoleacetic acid [5‐HIAA]) were quantified in the brain. The system had a detection limit of 0.1 pg/sample for all monoamines.

### 2.5 Analysis of the fecal microbiome

DNA was extracted from fecal samples using a commercial QuickGene DNA tissue kit (DT‐S, Kurabo, Osaka, Japan), as previously reported (Inoue et al., 2016; Tsukahara et al., 2018). The extracted DNA was subjected to a polymerase chain reaction (PCR) targeting the V3–V4 region of the bacterial 16S rRNA gene using primers 341F (5'‐ TCGTCGGCAGCGTCAGATGTGTATAAGAGACAGCCTACGGGNGGCWGCAG‐3') and 805R (5'‐GTCTCGTGGGCTCGGAGATGTGTATAAGAGACAGGACTACHVGGGTATCTAATCC‐3'). The applied PCR conditions were as follows: 1 cycle at 95°C for 3 min, 25 cycles of denaturation 95°C for 30 s, annealing at 55°C for 30 s and extension at 72°C for 30 s, and 1 final cycle at 72°C for 5 min. Electrophoresis was then performed on the resultant amplicons to confirm that the specimen was PCR‐amplified, and then purified using NucleoFast 96 (U3100B; Takara Bio, Shiga, Japan). A second PCR was conducted using the KAPA Hifi Hotstart Ready Mix (KK2602; Kapa Biosystems, Wilmington, MA, USA) to attach a unique combination of dual indices (indices I5 and I7). The resultant PCR product was then purified using SequalPrep Normalization Plate Kit (A1051001; Life Technologies, Tokyo, Japan). Each of the normalized amplicon was then evenly pooled and concentrated using AMPure XP beads (A63881; Beckman Coulter, Tokyo, Japan). Final DNA concentrations were confirmed by KAPA Library Quantification Kits (KK4828; Kapa Biosystems). The resulting libraries were sequenced using an Illumine Miseq platform with Miseq Reagent Kit v3 (MS‐102‐3003; Illumina). The obtained sequences were filtered using the bowtie‐2 program (version 2–2.2.4) to remove reads mapped to the PhiX174 sequence. Low‐quality reads, defined as those with a PHRED quality score of less than 17, a length of less than 150 bp, and an average quality score of less than 25, were excluded. Paired‐end reads that passed the quality filters were combined, and the resulting sequences were analyzed using the Quantitative Insights into Microbial Ecology 2 (QIIME2) (Bolyen et al., 2019). Sequences were assigned to operational taxonomic units (OTUs) using open‐reference OTU selection with a 97% pairwise identity threshold and SILVA reference database. The minimum read count after filtering was 19,973, with a median read count of 34,772.

### 2.6 Statistical analysis

Continuous data are presented as mean ± standard deviation (SD), whereas non‐normally distributed parameters, as determined by the Shapiro–Wilk normality test, are reported as median with interquartile range (IQR). Statistical analyses of all animal experiments were performed using the JMP PRO v.17 software package for Windows (SAS Institute, Japan).

Repeated‐measures analysis of variance (ANOVA) was employed to compare body weight and food intake between the CON and KES groups. Behavioral parameters and brain monoamine levels in the groups that received diets with or without KES were evaluated using a two‐way ANOVA. The relative abundance of the gut microbiota was assessed using the Kruskal–Wallis test, and when the *p*‐values were lower than the Bonferroni‐corrected threshold based on the total number of tests, the Steel–Dwass test was applied to determine significant differences between the two indicated groups. Associations between the total striatal DA levels and distance measured in the OF test, as well as between striatal DA levels and the relative abundance of bacterial species, were examined using Spearman's rank correlation coefficients.

For the generation of relative abundance plots and calculation of α‐diversity metrics (Shannon index, Chao 1, and observed species) and β‐diversity parameters (weighted and unweighted UniFrac metrics), the QIIME2 was used. Principal coordinate analysis (PCoA) plots were created using the unweighted UniFrac method and visualized as 3D graphs using QIIME2. The α‐diversity of the gut microbiota was assessed using two‐way ANOVA. As the *p*‐values were lower than the Bonferroni‐corrected threshold based on the total number of tests, the Tukey–Kramer's test was applied to determine significant differences between the two indicated groups. To evaluate the differences in bacterial composition among different groups of mice based on β‐diversity, unweighted UniFrac distances from each group to G1‐CON were evaluated using the Steel test. Moreover, permutational multivariate ANOVA (PERMANOVA) was also conducted using the Adonis function in the vegan package in R Studio using R 3.6.2.

## 3 RESULTS

### 3.1 KES supplementation had no effect on body weight gain

**FIGURE 1:**
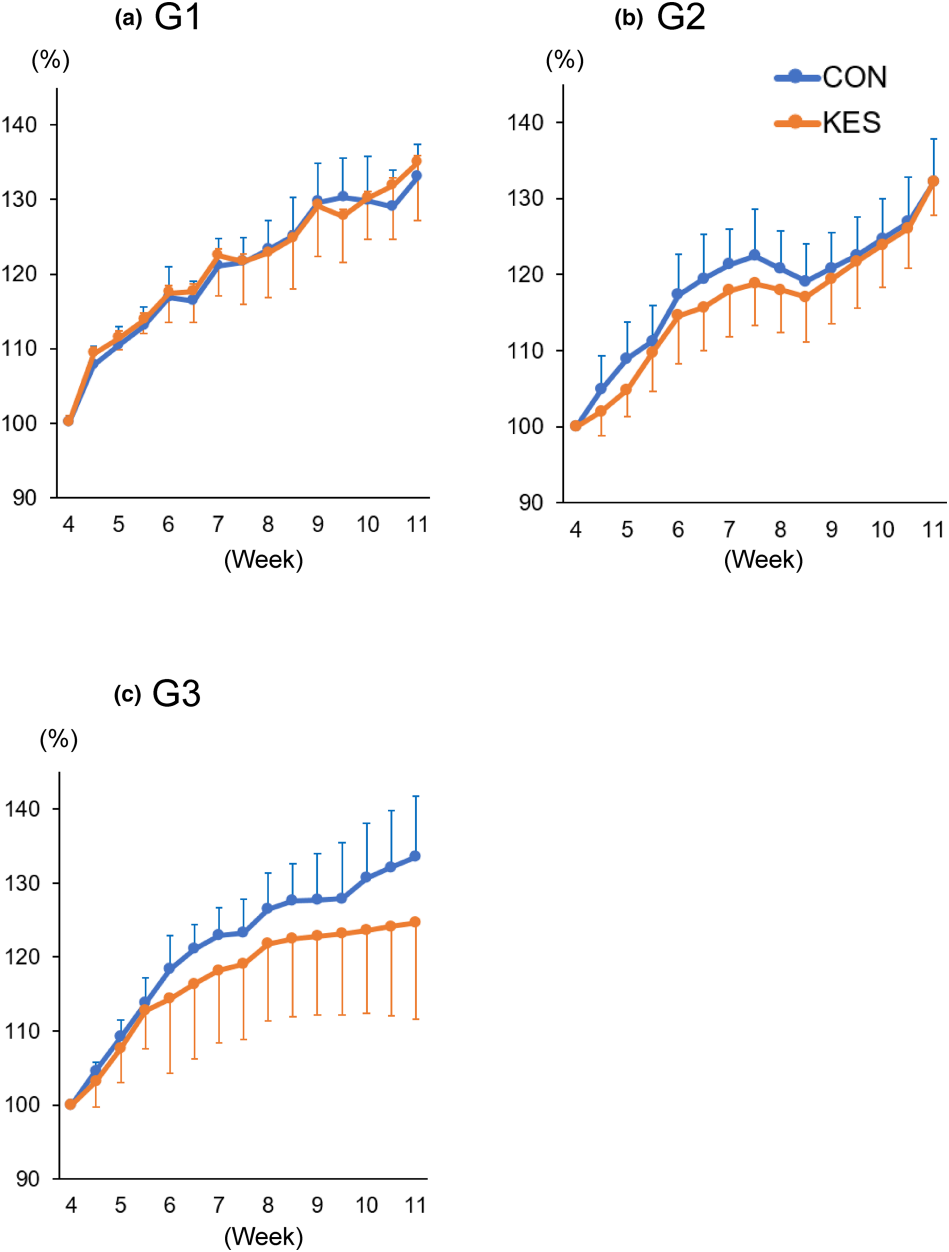
Effects of KES administration on body weight. After weaning, body weight in the CON and KES groups (*n* = 12) of the first generation (G1), (a), second generation (G2), (b), or third generation (G3), (c) was measured twice a week for seven consecutive weeks. The CON and KES mice at 4 weeks of age weighed 20 ± 0.7 and 22.8 ± 1.2 g in G1, 22.1 ± 1.6 and 22.5 ± 0.9 g in G2, and 20.8 ± 1.2 and 21 ± 2.1 g in G3. All data are expressed as mean ± SD. CON, control; group fed control diet without KES supplementation; KES, group fed KES‐supplemented diet.

As shown in Figure 1, repeated‐measures ANOVA did not show a significant difference in the percentage of body weight relative to the respective basal weight between the CON and KES groups at any generation (G1, F_(1,22)_ = 0.12, *p* = 0.73; G2, F_(1,22)_ = 1.49, *p* = 0.24; G3, F_(1,22)_ = 2.59, *p* = 0.12). Similarly, there was no difference in food consumption between the CON and KES groups at any generations (G1, F_(1,22)_ = 1.36, *p* = 0.25; G2, F_(1,22)_ = 1.59, *p* = 0.22; G3, F_(1,22)_ = 0.56, *p* = 0.46).

### 3.2 KES‐supplemented diets induce behavioral changes

**FIGURE 2:**
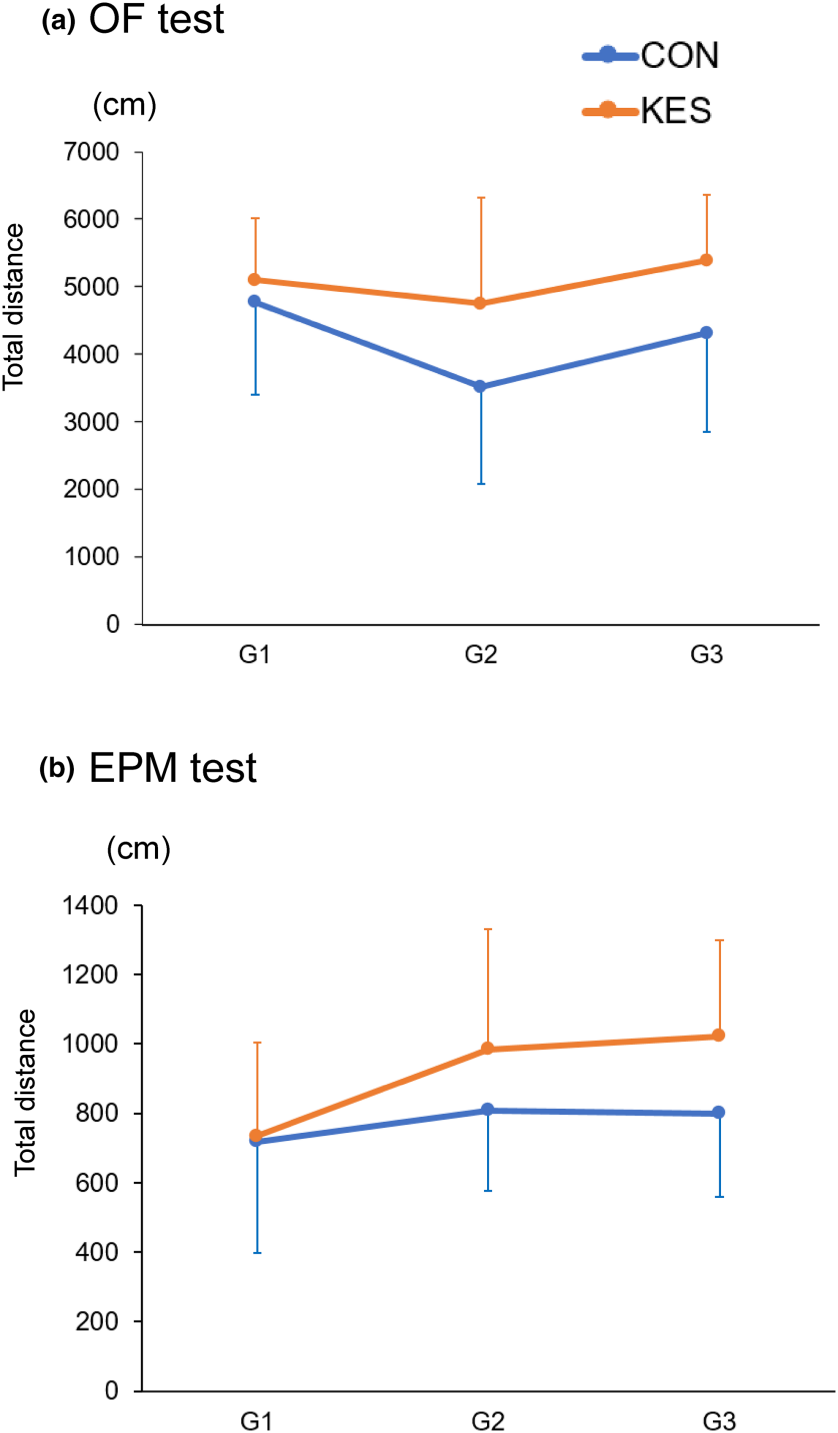
Dietary KES supplementation induces behavioral alterations. Total distance was evaluated as a parameter of locomotor activity using OF (a) and EPM (b) tests (*n* = 12 per generation for the CON and KES groups). Vertical bar indicates the total distance (cm) traveled during 20 min. G1, G2, and G3 indicate the first, second, and third generations, respectively. CON, control; group fed control diet without KES supplementation; EPM, elevated plus‐maze; KES, group fed KES‐supplemented diet; OF, open‐field.

As summarized in Table 1 and Figure 2, the two‐way ANOVA results revealed significant effects of diet on the total distances traveled. Locomotor activity was higher in the KES group than in the CON group when evaluated using OF (Figure 2a, F_(1,66)_ = 8.02, *p* = 0.0061) and EPM (Figure 2b, F_(1,66)_ = 4.25, *p* = 0.0433) tests. Generations significantly affected immobility time, as measured by the FS tests (F_(2,66)_ = 4.65, *p* = 0.0129). In contrast, no significant effects of diet or generation were observed for any other behavioral parameters (Table 1).

**TABLE 1:** Behavioral characteristics in CON and KES groups.

Diets	CON			KES			Two‐way ANOVA		
Generations	G1	G2	G3	G1	G2	G3			
	Mean (SD)			Mean (SD)			Diet	Generation	Interaction
Time spent in peripheral area (min)	1154.64 (60.39)	1152.41 (58.58)	1152.13 (71.13)	1139.81 (67.3)	1154.64 (55.8)	1168.88 (21.21)	0.9198	0.7317	0.643
Total distance traveled (OF) (cm)	4772.46 (1368.69)	3513.18 (1426.6)	4315.54 (1458.91)	5094.17 (913.03)	4745.74 (1575.38)	5385.45 (974.39)	**0.0061**	0.0724	0.4428
Time spent in closed arms (min)	130.48 (107.65)	93.48 (67.01)	103.79 (104.45)	118.31 (97.42)	79.21 (59.22)	151.97 (106.34)	0.7405	0.2329	0.4184
Total distance traveled (EPM) (cm)	719.36 (322.23)	808.57 (230.76)	799.38 (240.18)	735.42 (269.86)	984.1 (347.62)	1022.09 (276.99)	**0.043**	0.0524	0.4236
Immobility time (min)	84.08 (45.1)	115 (32.45)	136.58 (57.96)	76.88 (40.93)	125.67 (65.95)	94.08 (46.13)	0.2671	**0.0129**	0.1725

*Note*: Total distance traveled was measured as a parameter of locomotor activity using OF and EPM tests (*n* = 12 per each group). Anxiety‐like behaviors were evaluated using total time spent in peripheral areas during OF test or total time spent in the closed arm during EPM test. Depressive‐like behaviors were assessed based on total time immobilized in FS test (*n* = 12 per each group). Two‐way ANOVA was conducted to examine the effects of diets, generations, and their interaction on behavioral variables for each behavior. Bold figures indicate a significant effect of the factor on the indicated behavior.

Abbreviations: CON, group fed a control diet without KES supplementation; EPM, elevated plus‐maze; FS, forced swimming; G1, first generation; G2, second generation; G3, third generation; KES, group fed a KES‐supplemented diet; OF, open‐field.

### 3.3 KES exerts a significant effect on monoamine concentrations in various regions of the brain

As summarized in Table 2, diet significantly affected 5‐HT, DA, and DOPAC levels in the striatum (5‐HT, F_(1,64)_ = 9.16, *p* = 0.0035; DA, F_(1,63)_ = 11.61, *p* = 0.0011; DOPAC, F_(1,64)_ = 6.36, *p* = 0.0141). In contrast, generation had a significant effect on striatal 5‐HIAA and DA levels (5‐HIAA, F_(1,64)_ = 8.72, *p* = 0.0004; DA, F_(1,63)_ = 12.14, *p* < 0.0001). Notably, a significant interaction between diet and the generation of striatal DA levels (F_(1,63)_ = 3.49, *p* = 0.0364) was found, indicating that diet modulated how the striatal DA system responded to generational change.

**TABLE 2:** Levels of monoamines and their metabolites in various regions of the brain.

	CON			KES			ANOVA		
	G1	G2	G3	G1	G2	G3			
	Mean (SD)			Mean (SD)			Diet	Generation	Interaction
Striatum									
5‐HT	610.9 (224.6)	839.3 (528.4)	689.5 (269.7)	1091.2 (1150.3)	899.3 (618.8)	1617.4 (867.1)	**0.0035**	0.2363	0.0953
5‐HIAA	2394.6 (1327.2)	1283.1 (729.4)	800.1 (427.2)	3398 (3268.3)	1442.5 (871.4)	1553.9 (1058)	0.086	**0.0004**	0.6239
DA	1049.6 (1013.6)	1502.2 (1211.1)	2225.1 (1396.9)	1752.9 (2184.7)	2057 (1702.3)	5098.9 (2277)	**0.0011**	**<0.0001**	**0.0364**
DOPAC	1589.2 (1180)	1175.1 (995.7)	1316.7 (785.4)	2672.2 (2421.4)	2140.6 (2808.4)	2385.2 (1253.8)	**0.0141**	0.6462	0.9919
E	1706.2 (1134.2)	1033.4 (703.8)	975 (1340.3)	5058.3 (10870.7)	1030.5 (743.4)	1804.5 (2282.1)	0.2043	0.1715	0.4271
MHPG	276.8 (128.8)	160.4 (83.1)	138.7 (53.3)	394.6 (519.5)	166.6 (60.4)	2145.4 (6694.7)	0.283	0.4299	0.378
Frontal cortex									
5‐HT	178.4 (100.2)	337.2 (122.6)	314.5 (129.4)	224.5 (109.5)	245.2 (99.5)	358.2 (145)	0.9804	**0.0008**	0.0779
5‐HIAA	829.8 (318.3)	817.9 (399)	615.8 (223.5)	1060.2 (243.9)	804.7 (433.1)	665.3 (336.9)	0.2634	0.0095	0.4286
DA	69.8 (34.6)	99.4 (39.1)	85.2 (43.5)	71 (21.3)	75.4 (32.7)	133.8 (190.5)	0.6666	0.2781	0.3215
DOPAC	62.3 (20.6)	77.3 (32.3)	58.8 (23.6)	86.5 (29.3)	85.6 (75.3)	79 (99.6)	0.1844	0.7362	0.8745
NE	223.4 (82.8)	341.7 (102.4)	339.4 (120.6)	274.6 (93.2)	270.7 (89.4)	300.5 (89.8)	0.3954	**0.0329**	0.086
MHPG	123.9 (57.1)	124.5 (72.3)	98.6 (48.9)	132.1 (40.8)	116 (92.3)	100.1 (49.7)	0.9782	0.2669	0.8981
Brain stem									
5‐HT	429.9 (171.3)	513.3 (205.5)	539 (112.3)	288.4 (156)	528.3 (523.6)	656.1 (167.7)	0.9592	**0.0081**	0.2335
5‐HIAA	1307.4 (385.5)	808.3 (179.1)	995.9 (260.8)	1230.1 (424.6)	1005 (620.9)	950.8 (302.5)	0.7877	**0.0044**	0.4151
DA	127.1 (47.7)	164.5 (65.7)	215.5 (68.6)	115.9 (33.5)	216.9 (148.6)	162.6 (33.9)	0.8316	**0.0031**	0.0656
DOPAC	69.2 (19.6)	56 (17.6)	70.1 (33.9)	57.5 (24.2)	87.4 (63.1)	53.7 (10.9)	0.8885	0.5412	**0.0267**
NE	535.3 (174.9)	631.5 (118.5)	594.6 (46.3)	414.5 (149.2)	684.5 (628.8)	618.5 (98.8)	0.8261	0.074	0.5215
MHPG	127.5 (35.9)	84.1 (25.8)	89.6 (11.8)	107.9 (41.2)	108.2 (80.1)	80.6 (11.9)	0.8767	**0.0268**	0.1722
Hippocampus									
5‐HT	313 (74.4)	543.7 (201.2)	489.3 (164.4)	368.7 (136.2)	502.4 (50.1)	539.2 (157.7)	0.6014	**0.0008**	0.5552
5‐HIAA	792 (182.7)	615.9 (183.4)	593 (177)	945.8 (272.7)	585.9 (152.8)	717.3 (166.3)	0.1455	**0.0007**	0.3603
DA	52.4 (15.8)	56.1 (27.6)	52.6 (28.1)	61.4 (19.2)	49.6 (20.8)	74.7 (23.5)	0.2556	0.4275	0.2353
DOPAC	29.2 (8.2)	42.6 (15.7)	36.7 (16.7)	40.4 (14.1)	33.3 (18.6)	37.9 (14.3)	0.8142	0.8339	0.1961
NE	258.3 (43.8)	331.7 (109.9)	310.3 (110)	293.3 (106.6)	296.2 (30.8)	361.3 (107.4)	0.5266	0.1824	0.3701
MHPG	89.1 (30.8)	80.3 (28.3)	73.2 (24.9)	98.8 (35.6)	70.4 (21.4)	78.4 (21.2)	0.8325	0.1042	0.5803

*Note*: All data (*ng*/*g*, *n* = 8–12 per group) are expressed as the mean (SD). Two‐way ANOVA was conducted to examine the effects of diets, generations, and their interaction on monoamine and metabolite levels for each brain region. Bold figures indicate a significant effect of the factor on the indicated behavior.

Abbreviations: 5‐HT, 5‐hydroxytryptamine; 5‐HIAA, 5‐hydroxyindoleacetic acid; CON, group fed a control diet without KES supplementation; DA, dopamine; DOPAC, 3,4‐dihydroxyphenylacetic acid; G1, first generation; G2, second generation; G3, third generation; KES, group fed a KES‐supplemented diet; MHPG, 3‐methoxy‐4‐hydroxyphenylglycol; NE, norepinephrine.

In other brain regions, diet had no significant impact on monoamines and their metabolites; in contrast, generation exerted significant effects on 5‐HT (F_(1,65)_ = 8.02, *p* = 0.0008) and NE (F_(1,65)_ = 3.60, *p* = 0.0329) in the frontal cortex, 5‐HT (F_(1,65)_ = 5.19, *p* = 0.0044), 5‐HIAA (F_(1,65)_ = 5.90, *p* = 0.0081), DA (F_(1,65)_ = 6.30, *p* = 0.0031), and MHPG (F_(1,65)_ = 3.83, *p* = 0.0268) in the brain stem, and 5‐HT (F_(1,41)_ = 8.52, *p* = 0.0008) and 5‐HIAA (F_(1,65)_ = 8.61, *p* = 0.0007) in the hippocampus. Only DOPAC levels in the brain stem (F_(1,65)_ = 3.83, *p* = 0.0267) showed a significant interaction between diet and age.

### 3.4 Striatal DA levels correlate with locomotor activities in the mice fed KES‐supplemented diets

Next, we used Pearson's correlation coefficients to examine the relationship between brain monoamines and behavioral characteristics.

Striatal DA levels were significantly correlated with locomotor activity measured using OF tests in the KES group but not in the CON group (KES, *r* = 0.4121, *p* = 0.0154; CON, *r* = 0.2384, *p* = 0.1615).

### 3.5 KES‐supplemented diets alter gut microbial diversities

**FIGURE 3:**
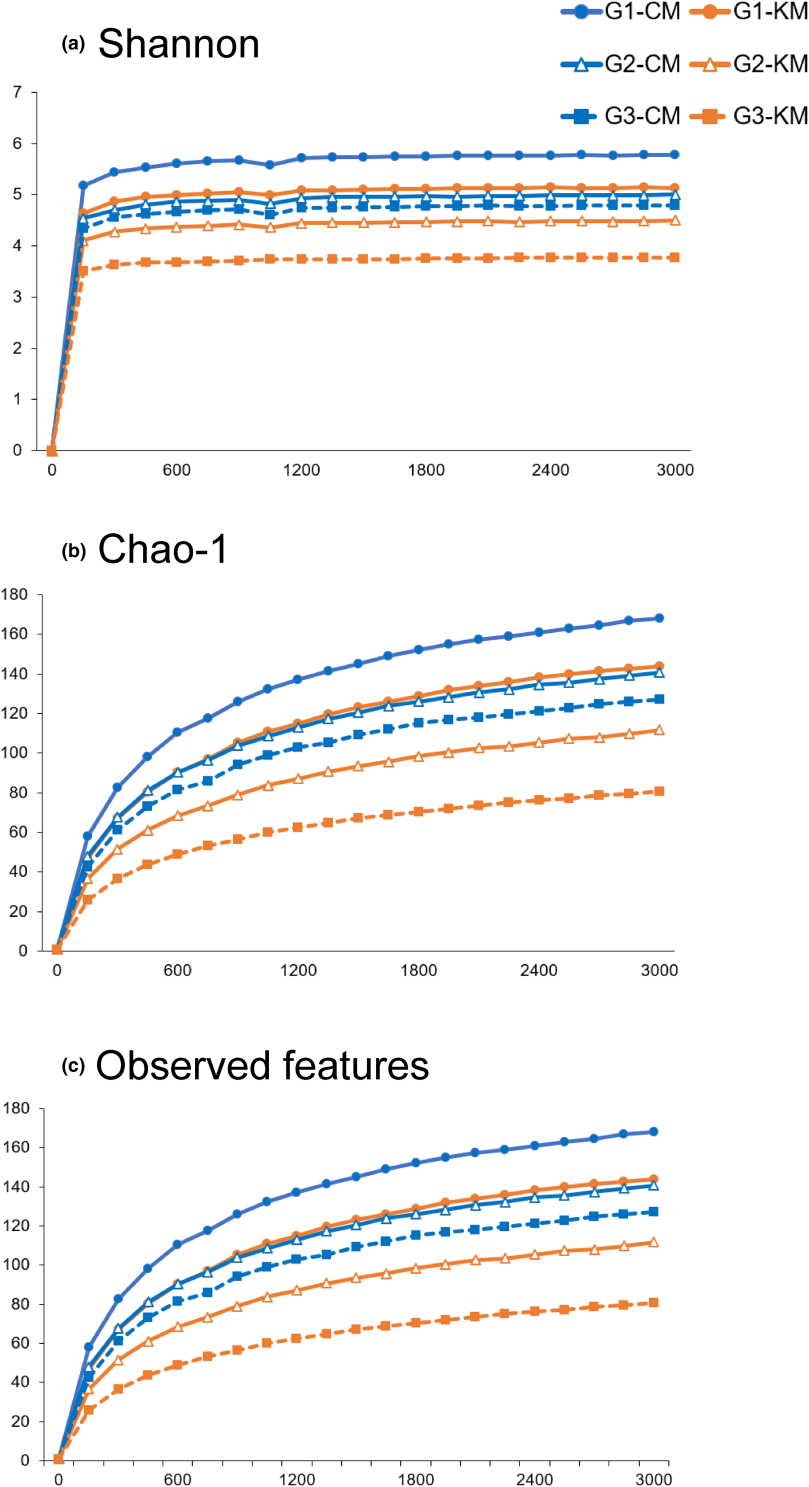
Effects of KES on microbial α‐diversities. (a) Chao‐1 values, (b) Shannon indices, and (c) observed features in the G1, G2, or G3 generation of CON and KES groups are shown as a parameter of α‐diversity. The horizontal axis indicates the number of sequence reads. All data (*n* = 12 per group) are mean ± SD.

**FIGURE 4:**
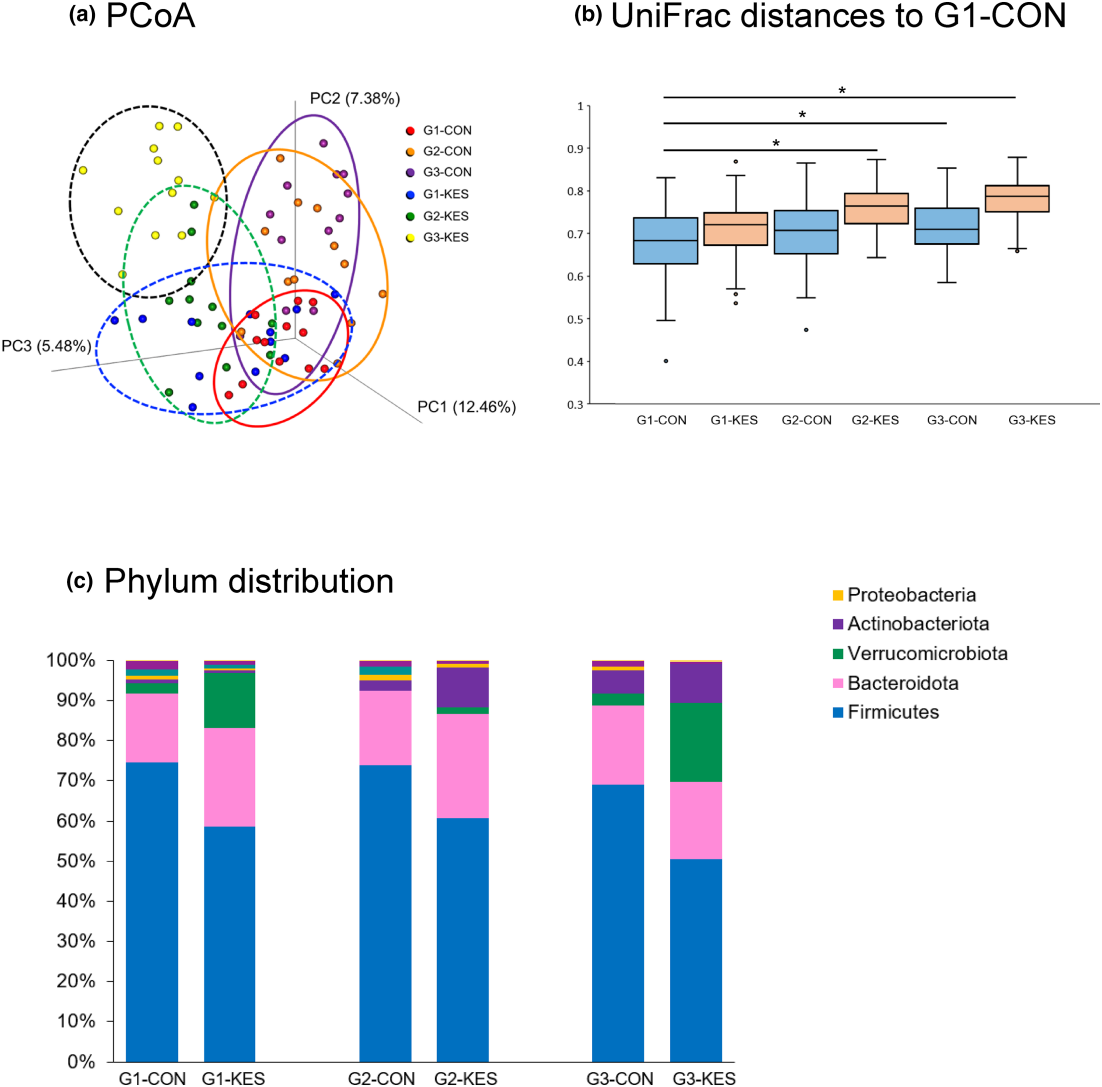
Effects of KES on microbial β‐diversities and taxonomic distribution. (a) The PCoA shows unweighted UniFrac distances for each group of mice (*n* = 12 per group). Each colored ellipse covers 95% of the samples belonging to a cluster. (b) Unweighted UniFrac distances to the G1 CON are visualized as box plots with medians (middle lines), first and third quartiles (box boundaries), and 1.5 times interquartile range (whiskers). An asterisk indicates a significant difference between the two indicated groups when evaluated using the Steel test (G1‐CON vs. G2‐KES, *p* < 0.0001; G1‐CON vs. G3‐CON, *p* = 0.0113; G1‐CON vs. G3‐KES, *p* < 0.0001). (c) Bar diagraphs depict the taxonomic distribution within each group of mice at the phylum level. CON, control; group fed control diet without KES supplementation; KES, group fed KES‐supplemented diet.

As summarized in Table 3 and Figure 3, the two‐way ANOVA showed significant effects of diet and generation on α‐diversities such as Shannon indices (F_(1,65)_ = 58.53, *p* < 0.0001; Generations, F_(1,65)_ = 54.34, *p* < 0.0001), Chao‐1 values (F_(1,65)_ = 26.64, p < 0.0001; Generations, F_(1,65)_ = 29.14, *p* < 0.0001), and observed features (Diet, F_(1,65)_ = 53.2, *p* < 0.0001; Generations, F_(1,65)_ = 43.34, *p* < 0.0001). The α‐diversity was significantly lower in the KES group than in the CON group. In addition, both groups had a significant reduction in α‐diversity across the three generations. Regarding β‐diversity, PCoA using unweighted UniFrac distances for each group of mice revealed a significantly different profile between the groups (Figure 4a). This was confirmed using PERMANOVAs, which showed a significant difference between the six groups (*f* = 6.99, *p* < 0.001). The KES group in the third generation showed the greatest difference in unweighted UniFrac distances compared to the CON group in the first generation (Figure 4b, *p* < 0.001).

**TABLE 3:** Alpha diversities in CON and KES groups.

	CON			KES			ANOVA		
	G1	G2	G3	G1	G2	G3			
	Mean (SD)			Mean (SD)			Diet	Generation	Interaction
Observed features	167.83 (26.75)	138.58 (20.40)	127.42 (20.21)	144.42 (18.24)	110.17 (13.87)	81.25 (10.05)	**<0.0001**	**<0.0001**	0.1009
Shannon entropy	5.79 (0.29)	4.98 (0.48)	4.79 (0.48)	5.14 (0.42)	4.49 (0.32)	3.77 (0.33)	**<0.0001**	**<0.0001**	0.0646
Chao1	203.55 (43.37)	175.70 (36.45)	151.88 (25.18)	180.33 (26.45)	140.97 (20.29)	102.92 (13.94)	**<0.0001**	**<0.0001**	0.319

	G1 (CON vs. KES)	G2 (CON vs. KES)	G3 (CON vs. KES)	CON (G2 vs. G1)	CON (G3 vs. G1)	KES (G2 vs. G1)	KES (G3 vs. G1)
Observed features	**0.0403**	**0.0063**	**<0.0001**	**0.0045**	**<0.0001**	**0.0005**	**<0.0001**
Shannon entropy	**0.0024**	**0.0426**	**<0.0001**	**<0.0001**	**<0.0001**	**0.0019**	**<0.0001**
Chao1	0.3864	0.0541	**0.0016**	0.1975	**0.0007**	**0.0191**	**<0.0001**

*Note*: All data (*n* = 12 per group) of upper table are expressed as the mean (SD). Data of lower table indicate *p*‐values calculated using the Tukey–Kramer's test. When two‐way ANOVAs were significantly different between the CON and KES groups, the Tukey–Kramer's test was applied to evaluate differences in the level of alpha diversities between the two indicated groups. Bold figures indicate a significant difference between the two indicated groups.

Abbreviations: CON, control; KES, group fed a KES‐supplemented diet.

Taxonomic analysis revealed a significant difference in the relative abundances of some phyla between the KES and CON groups (Figure 4c and Table S1). Regarding differences across generations, the relative percentage of the phylum *Actinobacteria* significantly increased in the KES group in the second and third generations relative to the first generation. Such an increase in the phylum *Actinobacteria* was also found in the CON group in the third generation compared to the CON group in the first generation. The KES group exhibited a significant decrease in the relative abundance of the phylum *Firmicutes* compared with the CON group throughout the different generations. Several bacterial genera showed significant differences between the KES and CON groups across different generations. Most genera identified in this study were lower in the KES group than in the CON group, whereas the genera *Bifidobacteria* and *Akkermansia* were higher in the KES group than in the CON group (Table S2). Similarly, at the species level, most species were lower in the KES group than in the CON group, whereas some species, including *Akkermansia muciniphila* and *Clostridiales*, were higher in the KES group than in the CON group (Table S3).

### 3.6 Correlations of striatal DA with α‐diversities and bacterial genera

Finally, we examined the relationship between striatal DA and bacterial composition.

As shown in Table 4, Spearman's rank‐order analysis revealed a significant negative correlation between the striatal DA levels and α‐diversity values (observed features, Shannon indices, and Chao‐1) in the KES group but not in the CON group. In addition, the genera *Enterococcus*, *Staphylococcus*, *Colidextribacter*, and UCG‐003 were negatively correlated with striatal DA levels in the KES group, although this correlation was not observed in the CON group (Table S4).

**TABLE 4:** Correlations between striatal DA and alpha diversities.

	CON		KES	
	Spearman *p*‐value	*p*‐value	Spearman *p*‐value	*p*‐value
Observed features	−0.274	0.105	−0.659	**2.25839E‐05**
Shannon	−0.34	0.043	−0.645	**3.87626E‐05**
Chao1	−0.243	0.153	−0.676	**1.139E‐05**

*Note*: Associations between striatal DA levels and alpha‐diversities were evaluated using Spearman's rank‐order test. Bold figures indicate a significant difference between the two indicated variables.

Abbreviations: CON, group fed a control diet without KES supplementation; DA, dopamine; KES, group fed a KES‐supplemented diet.

## 4 DISCUSSION

In the present study, mice fed KES‐supplemented diets for three consecutive generations were more active than those fed diets without KES. The KES group also exhibited increased brain DA and 5‐HT levels in the striatum. Such increased DA levels in the striatum were positively correlated with locomotor activity in the KES group but not in the CON group. Regarding the gut microbial ecology, α‐diversity (observed features, Shannon indices, and Chao‐1 values) was significantly lower in the KES group than in the CON group. Three‐dimensional PCoA revealed a significant difference between the KES and CON groups for each generation. Regarding the relative abundance at the genus level, most genera identified in this study were lower in the KES group than in the CON group. In contrast, the genera *Bifidobacteria* and *Akkermansia* in the KES group were higher than in the CON group. Spearman's rank‐order analysis showed significant negative correlations between striatal DA levels and α‐diversity values, as well as significant negative relationships between striatal DA levels and several genera in the KES group, whereas such correlations were not found in the CON group. Collectively, these results suggest that long‐term KES supplementation can induce an increase in locomotor activity concomitant with increased striatal DA levels, which may be related to KES‐induced changes in the gut microbiota.

In the current study, KES supplementation did not affect weight gain across the three generations, indicating that KES could be safely ingested over long periods without any apparent impact on physical development. Watanabe et al. (Watanabe, Tochio, et al., 2021) reported that rats fed diets containing 5% KES showed a significant decrease in serum insulin levels compared with rats fed a standard diet without KES, suggesting that KES may improve insulin sensitivity. In this study, whether KES supplementation could have an insulin resistance‐improving effect was unclear. Further studies are required to elucidate the effects of KES on the host metabolic functions.

In the present study, KES‐supplemented diets accelerated locomotor activity. Moreover, locomotor activity was correlated with striatal DA concentration in the KES group. The precise mechanism by which KES induces a change in behavioral phenotype remains to be clarified; however, Dohnalová et al. (Dohnalová et al., 2022) pointed to a potential gut–brain pathway that regulates exercise performance. They investigated the influence of the intestinal microbiome on brain circuits involved in exercise performance using comprehensive, deeply profiled, genetically, and metagenomically diverse mice maintained in gnotobiotic conditions. They demonstrated that the production of endocannabinoid metabolites in the gut, which is dependent on the microbiome, stimulated the activity of sensory neurons expressing the transient receptor potential cation channel subfamily V member 1, initiating an exercise‐induced afferent signal in the brain that ultimately led to the downregulation of monoamine oxidase expression in the striatum. This downregulation increased DA levels in the striatum and enhanced exercise capability. Thus far, we do not have any data on whether this microbiome‐derived endocannabinoid pathway is involved in the KES‐induced increase in locomotor performance; however, this is an intriguing question that should be answered in future research.

Dietary supplementation with KES positively influences the abundance and activity of *Bifidobacteria* in the gut (Koga et al., 2016; Shibata et al., 2009; Watanabe, Tochio, et al., 2021). This effect is attributed to the selective stimulation and utilization of KES by *Bifidobacteria*. Consistent with previous studies (Koga et al., 2016; Shibata et al., 2009; Watanabe, Tochio, et al., 2021), the genus *Bifidobacteria* was more abundant in the KES group than in the CON group in the current study. Notably, α‐diversity was significantly lower in the KES group than in the CON group, and the greatest difference between the two groups was observed in the third generation. In addition, a significant reduction in diversity was found throughout three consecutive generations in both groups of mice. Several factors can reduce gut microbial diversity (Bailey et al., 2011; Imhann et al., 2016; Jernberg et al., 2010; Sonnenburg et al., 2016; Yatsunenko et al., 2012). For example, antibiotics can kill some bacteria, decreasing overall microbial diversity (Jernberg et al., 2010). Diet is the most important factor that substantially affects gut microbial diversity (David et al., 2014; Moles & Otaegui, 2020; Sonnenburg et al., 2016). Generally, the more diverse the diet, the more diverse the microbiome and the more adaptable it is to perturbations (Heiman & Greenway, 2016). Chow diets exhibit greater heterogeneity than the purified diets employed in nutritional experiments, as they comprise macronutrients obtained from diverse sources such as whole wheat, dehulled soybean meal, ground corn, animal fat, and condensed whey (Pellizzon, 2016). Conversely, the diets employed in this study were significantly less diverse, such as AIN‐93G, which incorporates casein as the protein source, corn starch and sucrose as the carbohydrate sources, and corn oil as the exclusive fat source (Reeves et al., 1993). Therefore, the long‐term loss of dietary diversity may contribute to the emergence of reduced gut microbiome diversity. Further studies are needed to fully understand the mechanisms underlying the long‐lasting effects of dietary changes on gut microbial diversity over generations.

Behavioral changes induced by microorganisms have been observed in diverse host species (Ezenwa et al., 2012) and under specific circumstances. Notably, Toxoplasma infection has been well‐documented to alter mouse behavior (House et al., 2011; Vyas et al., 2007). Specifically, mice infected with Toxoplasma display insensitivity to the odor of cats, which are the end hosts of the parasites, rendering them more susceptible to predation. This phenomenon, known as “behavioral manipulation” or “mind control” (Sampson & Mazmanian, 2015; Thomas et al., 2005), facilitates the parasites' more efficient transmission to their definitive host via a series of orchestrated processes. Interestingly, Schnorr et al. (Schnorr et al., 2014) explored the comparison of gut bacteria and metabolites between Hadza hunter‐gatherers and Western populations. Their findings revealed greater bacterial diversity in the gut microbiota of Hadza individuals than in that of Westerners. This study suggests that the transition from a hunter‐gatherer to an agricultural lifestyle during the Neolithic era may have resulted in significant alterations in enteric bacterial composition due to drastic changes in diet. Therefore, it is interesting to speculate that such diet‐induced shifts in the gut microbiome may have contributed to an acquisition of social behavior (Sherwin et al., 2019). Although studies directly linking dietary differences, gut microbiota, and behavioral changes in animals are still limited, the current results using one of the prebiotics, KES, may be a useful animal model for clarifying this speculation. Further investigation into the specific mechanisms by which the gut microbiota influences behavior in different dietary niches would help deepen our understanding of the relationship between diet, gut microbiota, and behavior.

This study consists of several notable limitations that warrant discussion. First, one of the main limitations pertains to the methodology of substituting sucrose, in the dietary regimen, with KES. This substitution presents a substantive challenge in disentangling the precise effects of KES on host behaviors (i.e., particularly those related to reward mechanisms) and on the composition of the gut microbiota. Second, the evaluation of locomotor activity could studied more precisely through the use of metabolic cages, allowing for the assessment of energy expenditure in the context of 24‐h locomotor patterns. Lastly, our examination primarily focused on monoamine levels within select regions of the brain, with no concurrent exploration of relevant metabolites, proteins, or mRNA expression. Overall, these limitations can be used as a framework for the structuring of further, fine‐scale studies intended to validate findings brought forth through this study.

In conclusion, the long‐term administration of dietary KES across multiple generations resulted in modification of the host's locomotor activity, coinciding with elevated levels of striatal DA and reduced diversity in gut microbial populations. Enhanced functioning of the dopaminergic system in the striatum may be responsible for the observed increase in locomotor activity. Clearly, more refined and thorough research is required to explore the connection between transgenerational dietary effects and behavioral outcomes; however, such studies will enhance our understanding of the intricate interplay between gut microbiota, diet, and host behavior.

## AUTHOR CONTRIBUTIONS

Altanzul Altaisaikhan, Kazufumi Yoshihara, and Nobuyuki Sudo designed research; Altanzul Altaisaikhan, Kazufumi Yoshihara, Noriyuki Miyata, Yasunari Asano, Takafumi Suematsu, and Yoshihiro Kadota performed experiments; Altanzul Altaisaikhan, Kazufumi Yoshihara, Noriyuki Miyata, and Nobuyuki Sudo analyzed data; Altanzul Altaisaikhan, Kazufumi Yoshihara, and Nobuyuki Sudo interpreted results of experiments; Altanzul Altaisaikhan, Kazufumi Yoshihara, and Nobuyuki Sudo drafted manuscript; all authors read and approved the final version of manuscript.

## FUNDING INFORMATION

This work was supported by grants from JAPAN Society for the Promotion of Science (JP 20H04106 and JP23K18276: Nobuyuki Sudo), as well as by a Grant‐in‐Aid from the Smoking Research Foundation (2020‐192: Nobuyuki Sudo).

## ETHICS STATEMENT

Animal experiments were approved by the Ethics Committee on Animal Experiments of the Graduate School of Medical Sciences, Kyushu University (A20‐175‐0) and were conducted in compliance with the Guidelines for Animal Experiments of the Graduate School of Medical Sciences, Kyushu University, as well as the Law (No. 105) and Notification (No. 6) of the Japanese Government.

## Supporting information


Table S1.

Table S2.

Table S3.

Table S4.

Data S1.

## References

[phy215882-bib-0001] Asano, Y. , Hiramoto, T. , Nishino, R. , Aiba, Y. , Kimura, T. , Yoshihara, K. , Koga, Y. , & Sudo, N. (2012). Critical role of gut microbiota in the production of biologically active, free catecholamines in the gut lumen of mice. American journal of physiology. Gastrointestinal and liver physiology, 303, G1288–G1295. 10.1152/ajpgi.00341.2012 23064760

[phy215882-bib-0002] Bailey, M. T. , Dowd, S. E. , Galley, J. D. , Hufnagle, A. R. , Allen, R. G. , & Lyte, M. (2011). Exposure to a social stressor alters the structure of the intestinal microbiota: Implications for stressor‐induced immunomodulation. Brain, Behavior, and Immunity, 25, 397–407. 10.1016/j.bbi.2010.10.023 21040780 PMC3039072

[phy215882-bib-0003] Bolyen, E. , Rideout, J. R. , Dillon, M. R. , Bokulich, N. A. , Abnet, C. C. , al‐Ghalith, G. A. , Alexander, H. , Alm, E. J. , Arumugam, M. , Asnicar, F. , Bai, Y. , Bisanz, J. E. , Bittinger, K. , Brejnrod, A. , Brislawn, C. J. , Brown, C. T. , Callahan, B. J. , Caraballo‐Rodríguez, A. M. , Chase, J. , … Caporaso, J. G. (2019). Reproducible, interactive, scalable and extensible microbiome data science using QIIME 2. Nature Biotechnology, 37, 852–857. 10.1038/s41587-019-0209-9 PMC7015180 31341288

[phy215882-bib-0004] Carlson, A. L. , Xia, K. , Azcarate‐Peril, M. A. , Goldman, B. D. , Ahn, M. , Styner, M. A. , Thompson, A. L. , Geng, X. , Gilmore, J. H. , & Knickmeyer, R. C. (2018). Infant gut microbiome associated with cognitive development. Biological Psychiatry, 83, 148–159. 10.1016/j.biopsych.2017.06.021 28793975 PMC5724966

[phy215882-bib-0005] Clarke, G. , Grenham, S. , Scully, P. , Fitzgerald, P. , Moloney, R. D. , Shanahan, F. , Dinan, T. G. , & Cryan, J. F. (2013). The microbiome‐gut‐brain axis during early life regulates the hippocampal serotonergic system in a sex‐dependent manner. Molecular Psychiatry, 18, 666–673. 10.1038/mp.2012.77 22688187

[phy215882-bib-0006] Collins, S. M. , Surette, M. , & Bercik, P. (2012). The interplay between the intestinal microbiota and the brain. Nature Reviews. Microbiology, 10, 735–742. 10.1038/nrmicro2876 23000955

[phy215882-bib-0007] Cryan, J. F. , & Dinan, T. G. (2012). Mind‐altering microorganisms: The impact of the gut microbiota on brain and behaviour. Nature Reviews. Neuroscience, 13, 701–712. 10.1038/nrn3346 22968153

[phy215882-bib-0008] Cummings, J. H. (1981). Short chain fatty acids in the human colon. Gut, 22, 763–779. 10.1136/GUT.22.9.763 7028579 PMC1419865

[phy215882-bib-0009] Dabke, K. , Hendrick, G. , & Devkota, S. (2019). The gut microbiome and metabolic syndrome. The Journal of Clinical Investigation, 129, 4050–4057. 10.1172/JCI129194 31573550 PMC6763239

[phy215882-bib-0010] David, L. A. , Maurice, C. F. , Carmody, R. N. , Gootenberg, D. B. , Button, J. E. , Wolfe, B. E. , Ling, A. V. , Devlin, A. S. , Varma, Y. , Fischbach, M. A. , Dutton, R. J. , & Turnbaugh, P. J. (2014). Diet rapidly and reproducibly alters the human gut microbiome. Nature, 505, 559–563. 10.1038/nature12820 24336217 PMC3957428

[phy215882-bib-0011] Dohnalová, L. , Lundgren, P. , Carty, J. R. E. , Goldstein, N. , Wenski, S. L. , Nanudorn, P. , Thiengmag, S. , Huang, K.‐P. , Litichevskiy, L. , Descamps, H. C. , Betley, J. N. , & Thaiss, C. A. (2022). A microbiome‐dependent gut–brain pathway regulates motivation for exercise. Nature, 612, 739–747. 10.1038/s41586-022-05525-z 36517598 PMC11162758

[phy215882-bib-0012] Ezenwa, V. O. , Gerardo, N. M. , Inouye, D. W. , Medina, M. , & Xavier, J. B. (2012). Animal behavior and the microbiome. Science, 338, 198–199. 10.1126/science.1227412 23066064

[phy215882-bib-0013] Forsythe, P. , Sudo, N. , Dinan, T. , Taylor, V. H. , & Bienenstock, J. (2010). Mood and gut feelings. Brain, Behavior, and Immunity, 24, 9–16. 10.1016/j.bbi.2009.05.058 19481599

[phy215882-bib-0014] Gunn, R. K. , Huentelman, M. J. , & Brown, R. E. (2011). Are Sema5a mutant mice a good model of autism? A behavioral analysis of sensory systems, emotionality and cognition. Behavioural Brain Research, 225, 142–150. 10.1016/j.bbr.2011.07.008 21777623 PMC3170441

[phy215882-bib-0015] Hata, T. , Asano, Y. , Yoshihara, K. , Kimura‐Todani, T. , Miyata, N. , Zhang, X. T. , Takakura, S. , Aiba, Y. , Koga, Y. , & Sudo, N. (2017). Regulation of gut luminal serotonin by commensal microbiota in mice. PLoS One, 12, e0180745. 10.1371/journal.pone.0180745 28683093 PMC5500371

[phy215882-bib-0016] Hata, T. , Miyata, N. , Takakura, S. , Yoshihara, K. , Asano, Y. , Kimura‐Todani, T. , Yamashita, M. , Zhang, X.‐T. , Watanabe, N. , Mikami, K. , Koga, Y. , & Sudo, N. (2019). The gut microbiome derived from anorexia nervosa patients impairs weight gain and behavioral performance in female mice. Endocrinology, 160, 2441–2452. 10.1210/en.2019-00408 31504398

[phy215882-bib-0017] Heijtz, R. D. , Wang, S. , Anuar, F. , Qian, Y. , Bjorkholm, B. , Samuelsson, A. , Hibberd, M. L. , Forssberg, H. , & Pettersson, S. (2011). Normal gut microbiota modulates brain development and behavior. Proceedings of the National Academy of Sciences, 108, 3047–3052. 10.1073/pnas.1010529108 PMC3041077 21282636

[phy215882-bib-0018] Heiman, M. L. , & Greenway, F. L. (2016). A healthy gastrointestinal microbiome is dependent on dietary diversity. Molecular Metabolism, 5, 317–320. 10.1016/j.molmet.2016.02.005 27110483 PMC4837298

[phy215882-bib-0019] House, P. K. , Vyas, A. , & Sapolsky, R. (2011). Predator cat odors activate sexual arousal pathways in brains of toxoplasma gondii infected rats. PLoS One, 6, e23277. 10.1371/journal.pone.0023277 21858053 PMC3157360

[phy215882-bib-0020] Imhann, F. , Bonder, M. J. , Vich Vila, A. , Fu, J. , Mujagic, Z. , Vork, L. , Tigchelaar, E. F. , Jankipersadsing, S. A. , Cenit, M. C. , Harmsen, H. J. M. , Dijkstra, G. , & Franke, L. (2016). Proton pump inhibitors affect the gut microbiome. Gut, 65, 740–748. 10.1136/gutjnl-2015-310376 26657899 PMC4853569

[phy215882-bib-0021] Inoue, R. , Sakaue, Y. , Sawai, C. , Sawai, T. , Ozeki, M. , Romero‐Pérez, G. A. , & Tsukahara, T. (2016). A preliminary investigation on the relationship between gut microbiota and gene expressions in peripheral mononuclear cells of infants with autism spectrum disorders. Bioscience, Biotechnology, and Biochemistry, 80, 2450–2458. 10.1080/09168451.2016.1222267 27581276

[phy215882-bib-0022] Jernberg, C. , Löfmark, S. , Edlund, C. , & Jansson, J. K. (2010). Long‐term impacts of antibiotic exposure on the human intestinal microbiota. Microbiology, 156, 3216–3223. 10.1099/mic.0.040618-0 20705661

[phy215882-bib-0023] Kimura‐Todani, T. , Hata, T. , Miyata, N. , Takakura, S. , Yoshihara, K. , Zhang, X. T. , Asano, Y. , Altaisaikhan, A. , Tsukahara, T. , & Sudo, N. (2020). Dietary delivery of acetate to the colon using acylated starches as a carrier exerts anxiolytic effects in mice. Physiology & Behavior, 223, 113004. 10.1016/j.physbeh.2020.113004 32525009

[phy215882-bib-0024] Koga, Y. , Tokunaga, S. , Nagano, J. , Sato, F. , Konishi, K. , Tochio, T. , Murakami, Y. , Masumoto, N. , Tezuka, J. , Sudo, N. , Kubo, C. , & Shibata, R. (2016). Age‐associated effect of kestose on *Faecalibacterium* prausnitzii and symptoms in the atopic dermatitis infants. Pediatric Research, 80, 844–851. 10.1038/PR.2016.167 27537603 PMC5156669

[phy215882-bib-0025] Li, W. , Dowd, S. E. , Scurlock, B. , Acosta‐Martinez, V. , & Lyte, M. (2009). Memory and learning behavior in mice is temporally associated with diet‐induced alterations in gut bacteria. Physiology & Behavior, 96, 557–567. 10.1016/j.physbeh.2008.12.004 19135464

[phy215882-bib-0026] Martinez‐Gonzalez, M. A. , & Bes‐Rastrollo, M. (2014). Dietary patterns, Mediterranean diet, and cardiovascular disease. Current Opinion in Lipidology, 25, 20–26. 10.1097/MOL.0000000000000044 24370845

[phy215882-bib-0027] Moles, L. , & Otaegui, D. (2020). The impact of diet on microbiota evolution and human health. Is diet an adequate tool for microbiota modulation? Nutrients, 12, 1654. 10.3390/nu12061654 32498430 PMC7352211

[phy215882-bib-0028] Mozaffarian, D. , Hao, T. , Rimm, E. B. , Willett, W. C. , & Hu, F. B. (2011). Changes in diet and lifestyle and long‐term weight gain in women and men. The New England Journal of Medicine, 364, 2392–2404. 10.1056/NEJMOA1014296 21696306 PMC3151731

[phy215882-bib-0029] Neufeld, K. M. , Kang, N. , Bienenstock, J. , & Foster, J. A. (2011). Reduced anxiety‐like behavior and central neurochemical change in germ‐free mice. Neurogastroenterology and Motility, 23, 255–264. 10.1111/j.1365-2982.2010.01620.x 21054680

[phy215882-bib-0030] Nishino, R. , Mikami, K. , Takahashi, H. , Tomonaga, S. , Furuse, M. , Hiramoto, T. , Aiba, Y. , Koga, Y. , & Sudo, N. (2013). Commensal microbiota modulate murine behaviors in a strictly contamination‐free environment confirmed by culture‐based methods. Neurogastroenterology and Motility, 25, 521–528. 10.1111/nmo.12110 23480302

[phy215882-bib-0031] Pellizzon, M. (2016). Choice of laboratory animal diet influences intestinal health. Lab Animal, 45, 238–239. 10.1038/laban.1014 27203268

[phy215882-bib-0032] Popkin, B. M. , Adair, L. S. , & Ng, S. W. (2012). Global nutrition transition and the pandemic of obesity in developing countries. Nutrition Reviews, 70, 3–21. 10.1111/J.1753-4887.2011.00456.X 22221213 PMC3257829

[phy215882-bib-0033] Qin, J. , Li, R. , Raes, J. , Arumugam, M. , Burgdorf, K. S. K. S. , Manichanh, C. , Nielsen, T. , Pons, N. , Levenez, F. , Yamada, T. , Mende, D. R. , Li, J. , Xu, J. , Li, S. , Shengting, S. , Li, D. , Cao, J. , Wang, B. B. , Liang, H. , … Zoetendal, E. (2010). A human gut microbial gene catalogue established by metagenomic sequencing. Nature, 464, 59–65. 10.1038/nature08821 20203603 PMC3779803

[phy215882-bib-0034] Reeves, P. G. , Nielsen, F. H. , & Fahey, G. C., Jr. (1993). AIN‐93 purified diets for laboratory rodents: Final report of the American Institute of Nutrition ad hoc writing committee on the reformulation of the AIN‐76A rodent diet. The Journal of Nutrition, 123, 1939–1951. 10.1093/jn/123.11.1939 8229312

[phy215882-bib-0035] Sampson, T. R. , Debelius, J. W. , Thron, T. , Janssen, S. , Shastri, G. G. , Ilhan, Z. E. , Challis, C. , Schretter, C. E. , Rocha, S. , Gradinaru, V. , Chesselet, M. F. , Keshavarzian, A. , Shannon, K. M. , Krajmalnik‐Brown, R. , Wittung‐Stafshede, P. , Knight, R. , & Mazmanian, S. K. (2016). Gut microbiota regulate motor deficits and neuroinflammation in a model of Parkinson's disease. Cell, 167, 1469–1480.e12. 10.1016/j.cell.2016.11.018 27912057 PMC5718049

[phy215882-bib-0036] Sampson, T. R. , & Mazmanian, S. K. (2015). Control of brain development, function, and behavior by the microbiome. Cell Host & Microbe, 17, 565–576. 10.1016/j.chom.2015.04.011 25974299 PMC4442490

[phy215882-bib-0037] Schnorr, S. L. , Candela, M. , Rampelli, S. , Centanni, M. , Consolandi, C. , Basaglia, G. , Turroni, S. , Biagi, E. , Peano, C. , Severgnini, M. , Henry, A. G. , & Crittenden, A. N. (2014). Gut microbiome of the Hadza hunter‐gatherers. Nature Communications, 5, 3654. 10.1038/ncomms4654 PMC3996546 24736369

[phy215882-bib-0038] Sender, R. , Fuchs, S. , & Milo, R. (2016). Revised estimates for the number of human and bacteria cells in the body. PLoS Biology, 14, e1002533. 10.1371/journal.pbio.1002533 27541692 PMC4991899

[phy215882-bib-0039] Sherwin, E. , Bordenstein, S. R. , Quinn, J. L. , Dinan, T. G. , & Cryan, J. F. (2019). Microbiota and the social brain. Science, 366, eaar2016. 10.1126/science.aar2016 31672864

[phy215882-bib-0040] Shibata, R. , Kimura, M. , Takahashi, H. , Mikami, K. , Aiba, Y. , Takeda, H. , & Koga, Y. (2009). Clinical effects of kestose, a prebiotic oligosaccharide, on the treatment of atopic dermatitis in infants. Clinical and Experimental Allergy, 39, 1397–1403. 10.1111/j.1365-2222.2009.03295.x 19508323

[phy215882-bib-0041] Sonnenburg, E. D. , Smits, S. A. , Tikhonov, M. , Higginbottom, S. K. , Wingreen, N. S. , & Sonnenburg, J. L. (2016). Diet‐induced extinctions in the gut microbiota compound over generations. Nature, 529, 212–215. 10.1038/nature16504 26762459 PMC4850918

[phy215882-bib-0042] Statovci, D. , Aguilera, M. , Macsharry, J. , & Melgar, S. (2017). The impact of western diet and nutrients on the microbiota and immune response at mucosal interfaces. Frontiers in Immunology, 8, 838. 10.3389/fimmu.2017.00838 28804483 PMC5532387

[phy215882-bib-0043] Sudo, N. , Chida, Y. , Aiba, Y. , Sonoda, J. , Oyama, N. , Yu, X.‐N. , Kubo, C. , & Koga, Y. (2004). Postnatal microbial colonization programs the hypothalamic‐pituitary‐adrenal system for stress response in mice. The Journal of Physiology, 558, 263–275. 10.1113/jphysiol.2004.063388 15133062 PMC1664925

[phy215882-bib-0044] Suemaru, K. , Yasuda, K. , Cui, R. , Li, B. , Umeda, K. , Amano, M. , Mitsuhashi, H. , Takeuchi, N. , Inoue, T. , Gomita, Y. , & Araki, H. (2006). Antidepressant‐like action of nicotine in forced swimming test and brain serotonin in mice. Physiology & Behavior, 88, 545–549. 10.1016/j.physbeh.2006.05.007 16766001

[phy215882-bib-0045] Suzuki, N. , Aiba, Y. , Takeda, H. , Fukumori, Y. , & Koga, Y. (2006). Superiority of 1‐kestose, the smallest fructo‐oligosaccharide, to a synthetic mixture of fructo‐oligosaccharides in the selective stimulating activity on bifidobacteria. Bioscience and Microflora, 25, 109–116. 10.12938/BIFIDUS.25.109

[phy215882-bib-0046] Thomas, F. , Adamo, S. , & Moore, J. (2005). Parasitic manipulation: Where are we and where should we go? Behavioural Processes, 68, 185–199. 10.1016/j.beproc.2004.06.010 15792688

[phy215882-bib-0047] Tochio, T. , Kitaura, Y. , Nakamura, S. , Sugawa, C. , Takahashi, M. , Endo, A. , & Shimomura, Y. (2016). An alteration in the cecal microbiota composition by feeding of 1‐kestose results in a marked increase in the cecal butyrate content in rats. PLoS One, 11, e0166850. 10.1371/journal.pone.0166850 27861621 PMC5115820

[phy215882-bib-0048] Tomova, A. , Bukovsky, I. , Rembert, E. , Yonas, W. , Alwarith, J. , Barnard, N. D. , & Kahleova, H. (2019). The effects of vegetarian and vegan diets on gut microbiota. Frontiers in Nutrition, 1, 47. 10.3389/fnut.2019.00047 PMC6478664 31058160

[phy215882-bib-0049] Tsukahara, T. , Inoue, R. , Nakayama, K. , & Inatomi, T. (2018). Inclusion of bacillus amyloliquefaciens strain TOA5001 in the diet of broilers suppresses the symptoms of coccidiosis by modulating intestinal microbiota. Animal Science Journal, 89, 679–687. 10.1111/asj.12980 29282825

[phy215882-bib-0050] Vyas, A. , Kim, S.‐K. , Giacomini, N. , Boothroyd, J. C. , & Sapolsky, R. M. (2007). Behavioral changes induced by toxoplasma infection of rodents are highly specific to aversion of cat odors. Proceedings of the National Academy of Sciences of the United States of America, 104, 6442–6447. 10.1073/pnas.0608310104 17404235 PMC1851063

[phy215882-bib-0051] Walf, A. A. , & Frye, C. A. (2007). The use of the elevated plus maze as an assay of anxiety‐related behavior in rodents. Nature Protocols, 2, 322–328. 10.1038/nprot.2007.44 17406592 PMC3623971

[phy215882-bib-0052] Watanabe, A. , Kadota, Y. , Kamio, R. , Tochio, T. , Endo, A. , Shimomura, Y. , & Kitaura, Y. (2020). 1‐Kestose supplementation mitigates the progressive deterioration of glucose metabolism in type 2 diabetes OLETF rats. Scientific Reports, 10, 15674. 10.1038/s41598-020-72773-2 32973311 PMC7515885

[phy215882-bib-0053] Watanabe, A. , Tochio, T. , Kadota, Y. , Takahashi, M. , Kitaura, Y. , Ishikawa, H. , Yasutake, T. , Nakano, M. , Shinohara, H. , Kudo, T. , Nishimoto, Y. , Mizuguchi, Y. , Endo, A. , & Shimomura, Y. (2021). Supplementation of 1‐kestose modulates the gut microbiota composition to ameliorate glucose metabolism in obesity‐prone hosts. Nutrients, 13, 2983. 10.3390/NU13092983/S1 34578862 PMC8470827

[phy215882-bib-0054] Watanabe, N. , Mikami, K. , Hata, T. , Kimoto, K. , Nishino, R. , Akama, F. , Yamamoto, K. , Sudo, N. , Koga, Y. , & Matsumoto, H. (2021). Effect of gut microbiota early in life on aggressive behavior in mice. Neuroscience Research, 168, 95–99. 10.1016/j.neures.2021.01.005 33476684

[phy215882-bib-0055] Yatsunenko, T. , Rey, F. E. , Manary, M. J. , Trehan, I. , Dominguez‐Bello, M. G. , Contreras, M. , Magris, M. , Hidalgo, G. , Baldassano, R. N. , Anokhin, A. P. , Knight, R. , & Gordon, J. I. (2012). Human gut microbiome viewed across age and geography. Nature, 486, 222–227. 10.1038/nature11053 22699611 PMC3376388

[phy215882-bib-0056] Zhang, X. , Yoshihara, K. , Miyata, N. , Hata, T. , Altaisaikhan, A. , Takakura, S. , Asano, Y. , Izuno, S. , & Sudo, N. (2022). Dietary tryptophan, tyrosine, and phenylalanine depletion induce reduced food intake and behavioral alterations in mice. Physiology & Behavior, 244, 113653. 10.1016/j.physbeh.2021.113653 34800493

