# Supplementary material for: Dietary supplementation with 1‐kestose induces altered locomotor activity and increased striatal dopamine levels with a change in gut microbiota in male mice
Source: Physiol Rep. 2023 Dec 6;11(23):e15882. doi: 10.14814/phy2.15882 (PMC10698829; doi:10.14814/phy2.15882)
Supplement: Supplementary file 1 — Table S1. [file PHY2-11-e15882-s005.pdf]

**Supplementary Table 1. Relative abundance at phylum levels**

| | CON | | | KES | | | |
|---|---|---|---|---|---|---|---|
| | G1 | G2 | G3 | G1 | G2 | G3 | |
| | | Median (IQR) | | | Median (IQR) | | KW |
| d__Bacteria;p__Actinobacteriota | 0.77 (0.11) | 1.71 (2.3) | 5.25 (4.64) | 0.61 (0.41) | 10.32 (11.09) | 9.30 (4.51) | **< 0.0001** |
| d__Bacteria;p__Bacteroidota | 16.57 (5.73) | 17.79 (9.65) | 18.05 (7.68) | 24.36 (12.72) | 25.55 (11.82) | 19.67 (10.16) | 0.0287 |
| d__Bacteria;p__Firmicutes | 77.02 (10.85) | 75.54 (11.03) | 69.85 (16.19) | 57.60 (13.54) | 62.02 (9.35) | 48.87 (11.86) | **< 0.0001** |
| d__Bacteria;p__Proteobacteria | 0.22 (0.55) | 0.4 (1.33) | 0.34 (0.96) | 0.05 (0.33) | 0.04 (0.09) | 0 (0.03) | **0.0004** |
| d__Bacteria;p__Verrucomicrobiota | 0 (0.01) | 0 (0.01) | 0 (0.01) | 17.31 (19.9) | 0 (0) | 21.17 (18.52) | **< 0.0001** |

| | G1 (CON vs KES) | G2 (CON vs KES) | G3 (CON vs KES) | CON (G2 vs G1) | CON (G3 vs G1) | KES (G2 vs G1) | KES (G3 vs G1) |
|---|---|---|---|---|---|---|---|
| d__Bacteria;p__Actinobacteriota | 0.9965 | **0.0141** | 0.2833 | 0.2543 | **0.0141** | **0.0007** | **0.0005** |
| d__Bacteria;p__Bacteroidota | NT | | | | | | |
| d__Bacteria;p__Firmicutes | **0.0095** | **0.035** | **0.0011** | 0.9998 | 0.5298 | 0.9858 | 0.2271 |
| d__Bacteria;p__Proteobacteria | 0.9858 | 0.2262 | **0.0127** | 0.9323 | 0.999 | 0.8164 | **0.0123** |
| d__Bacteria;p__Verrucomicrobiota | **0.0318** | 0.953 | **0.0068** | 1 | 1 | **0.0226** | 0.6813 |

All data (n = 12 per group) are expressed as the median (interquartile range: IQR). Only phyla that are detected in more than 10 % samples were selected and processed for comparizons among the groups. When a p value was less than 0.01 in the KW test comparing the six groups, the Steel-Dwass test was applied to evaluate differences in the relative abundance between the indicated two groups.The p value of 0.01 in the KW test was calculated using the Bonferroni correction based on the total number of tests (5, $p = 0.05/5 = 0.01$). Bold figures indicate a significant difference between the indicated two groups ($p < 0.05$). G1, G2, and G3 show the 1st, 2nd, and 3rd generation, respectively. KW, Kruskal-Wallis test; CON, a group fed control diets; KES, a group fed 1-kestose-supplemented diets.
