# Supplementary material for: Dietary supplementation with 1‐kestose induces altered locomotor activity and increased striatal dopamine levels with a change in gut microbiota in male mice
Source: Physiol Rep. 2023 Dec 6;11(23):e15882. doi: 10.14814/phy2.15882 (PMC10698829; doi:10.14814/phy2.15882)
Supplement: Supplementary file 2 — Table S2. [file PHY2-11-e15882-s002.pdf]

Supplementary Table 2. Relative abundance at genus levels

	CON			KES										
	G1	G2 Median (IQR)	G3	G1	G2 Median (IQR)	G3	KW	G1 (CON vs KES)	G2 (CON vs KES)	G3 (CON vs KES)	CON (G2 vs G1)	CON (G3 vs G1)	KES (G2 vs G1)	KES (G3 vs G1)
d__Bacteria;p__Actinobacteriota;c__Actinobacteria;o__Bifidobacteriales;f__Bifidobacteriaceae;g__Bifidobacterium	0 (0)	0 (0.01)	4.05 (3.45)	0 (0.02)	**10.16 (10.8)**	8.59 (3.78)	**< 0.0001**	0.807	**0.0023**	0.1205	0.5014	**0.0032**	**0.0004**	**0.0004**
d__Bacteria;p__Actinobacteriota;c__Coriobacteriia;o__Coriobacteriales;__;__	0.03 (0.07)	0.01 (0.05)	0 (0.07)	0.02 (0.05)	0.02 (0.02)	0 (0.02)	0.5798	NT						
d__Bacteria;p__Actinobacteriota;c__Coriobacteriia;o__Coriobacteriales;f__Eggerthellaceae;__	0.74 (1.08)	1.63 (1.5)	0.92 (0.93)	0.59 (0.41)	**0.17 (0.22)**	**0.3 (0.33)**	**< 0.0001**	0.9858	**0.0009**	**0.0034**	0.2271	0.7857	**0.0141**	0.3811
d__Bacteria;p__Bacteroidota;c__Bacteroidia;o__Bacteroidales;f__Bacteroidaceae;g__Bacteroides	7.73 (3.07)	4.55 (3.52)	0.01 (2.99)	8.09 (5.19)	10.01 (6.71)	9.36 (12.09)	**0.0003**	0.9988	0.1381	0.373	**0.0294**	**0.0058**	0.9941	1
d__Bacteria;p__Bacteroidota;c__Bacteroidia;o__Bacteroidales;f__Muribaculaceae;g__Muribaculaceae	0 (0)	0 (0.03)	8.89 (10.94)	8.21 (4.77)	1.43 (3.46)	2.71 (4.01)	**< 0.0001**	**0.0001**	0.1365	0.491	0.4934	**0.0017**	**0.0116**	**0.0246**
d__Bacteria;p__Bacteroidota;c__Bacteroidia;o__Bacteroidales;f__Tannerellaceae;__	8.31 (4.31)	12.83 (15.96)	9.06 (3.28)	8.21 (4.44)	13.01 (5.72)	6.44 (8.05)	0.0235	NT						
d__Bacteria;p__Deferribacterota;c__Deferribacteres;o__Deferribacterales;f__Deferribacteraceae;g__Mucispirillum	0.54 (1.87)	0 (0.92)	0 (0)	0.01 (0.24)	0 (0)	0 (0)	**< 0.0001**	0.1287	0.4934	1	0.1792	**0.0005**	0.0763	0.0463
d__Bacteria;p__Firmicutes;c__Bacilli;o__Erysipelotrichales;f__Erysipelatoclostridiaceae;g__Candidatus_Stoquefichus	0.16 (0.3)	0 (0)	0 (0)	0.06 (0.1)	0 (0)	0 (0)	**< 0.0001**	0.3056	1	1	**0.0017**	**0.0017**	**0.0138**	**0.0138**
d__Bacteria;p__Firmicutes;c__Bacilli;o__Erysipelotrichales;f__Erysipelatoclostridiaceae;g__Erysipelatoclostridium	7.01 (4.25)	6.95 (5.61)	6.98 (4.53)	3.67 (2.49)	**1.77 (1.34)**	**2.37 (2.41)**	**< 0.0001**	0.2543	**0.0414**	**0.0042**	1	0.9858	0.0783	0.3811
d__Bacteria;p__Firmicutes;c__Bacilli;o__Erysipelotrichales;f__Erysipelotrichaceae;__	0.08 (0.15)	0 (0)	0 (0)	0.07 (0.09)	0 (0.05)	0 (0)	**< 0.0001**	0.998	0.8483	0.7365	**0.0186**	**0.0051**	0.273	**0.0052**
d__Bacteria;p__Firmicutes;c__Bacilli;o__Erysipelotrichales;f__Erysipelotrichaceae;g__Erysipelotrichaceae	0.06 (0.19)	0 (0)	0 (0)	0.03 (0.09)	0 (0)	0 (0)	**< 0.0001**	0.9996	0.9936	1	0.065	**0.0138**	**0.0226**	**0.0017**
d__Bacteria;p__Firmicutes;c__Bacilli;o__Erysipelotrichales;f__Erysipelotrichaceae;g__Faecalibaculum	0 (0)	17.6 (21.94)	23.8 (10)	0 (0)	20.13 (9.1)	25.17 (8.39)	**< 0.0001**	0.9424	0.9998	0.9998	**0.0001**	**0.0005**	**0.0002**	**0.0002**
d__Bacteria;p__Firmicutes;c__Bacilli;o__Erysipelotrichales;f__Erysipelotrichaceae;g__Turicibacter	0.18 (0.69)	0.09 (0.25)	0.24 (1.79)	**3.37 (2.47)**	0.14 (0.46)	**0 (0)**	**< 0.0001**	**0.022**	1	**0.0005**	0.9855	0.9826	**0.0074**	**0.0005**
d__Bacteria;p__Firmicutes;c__Bacilli;o__Erysipelotrichales;f__Erysipelotrichaceae;g__uncultured	0 (0)	0 (0)	0 (0)	0 (0.1)	**0.05 (0.09)**	0 (0.01)	**< 0.0001**	0.2766	**0.0005**	0.4934	0.9424	0.9424	0.8379	0.7105
d__Bacteria;p__Firmicutes;c__Bacilli;o__Lactobacillales;f__Enterococcaceae;g__Enterococcus	0.12 (0.6)	0.06 (0.24)	0 (0.04)	0.04 (0.06)	**0 (0)**	0 (0)	**< 0.0001**	0.6615	**0.0276**	0.6617	0.9323	**0.0328**	0.062	**0.0104**
d__Bacteria;p__Firmicutes;c__Bacilli;o__Lactobacillales;f__Lactobacillaceae;g__Lactobacillus	0.87 (1.05)	0.08 (0.4)	0 (0.31)	1.58 (2.1)	0.11 (0.48)	0 (0.65)	**< 0.0001**	0.4168	0.9531	0.9995	0.1175	**0.0044**	**0.0141**	**0.0373**
d__Bacteria;p__Firmicutes;c__Bacilli;o__Lactobacillales;f__Streptococcaceae;g__Lactococcus	1 (0.93)	0.79 (0.43)	0.72 (0.79)	0.33 (0.31)	**0.15 (0.09)**	**0.13 (0.3)**	**< 0.0001**	0.1575	**0.0007**	**0.0116**	0.9981	0.9858	0.4913	0.2543
d__Bacteria;p__Firmicutes;c__Bacilli;o__Lactobacillales;f__Streptococcaceae;g__Streptococcus	0.02 (0.05)	0 (0.02)	0.05 (0.05)	0 (0.02)	0 (0.03)	0.01 (0.02)	0.0306	NT						
d__Bacteria;p__Firmicutes;c__Bacilli;o__RF39;f__RF39;g__RF39	0.02 (0.15)	0 (0.01)	0 (0.05)	0.04 (0.12)	0.03 (0.05)	0 (0.03)	0.0276	NT						
d__Bacteria;p__Firmicutes;c__Bacilli;o__Staphylococcales;f__Staphylococcaceae;g__Staphylococcus	0.05 (0.17)	0.03 (0.07)	0 (0.03)	0.02 (0.02)	0 (0)	0 (0)	**0.0003**	0.6021	0.1952	0.3577	0.9822	0.2297	0.1884	**0.004**
d__Bacteria;p__Firmicutes;c__Clostridia;__;__;__	0.88 (0.9)	0.33 (0.94)	0.44 (0.65)	**0.17 (0.21)**	**0.07 (0.06)**	**0 (0.07)**	**< 0.0001**	**0.0064**	**0.0009**	**0.0028**	0.7329	0.3811	**0.0489**	**0.0082**
d__Bacteria;p__Firmicutes;c__Clostridia;o__Christensenellales;f__Christensenellaceae;__	0.03 (0.02)	0.01 (0.03)	0.04 (0.03)	0 (0.01)	0 (0.01)	**0 (0)**	**< 0.0001**	0.0539	0.6251	**0.0005**	0.3949	1	1	0.4934
d__Bacteria;p__Firmicutes;c__Clostridia;o__Christensenellales;f__Christensenellaceae;g__Christensenellaceae_R-7_group	0.06 (0.25)	0.19 (0.25)	0.11 (0.15)	0.05 (0.12)	**0 (0)**	**0 (0)**	**< 0.0001**	0.9993	**0.01**	**0.0124**	0.9904	0.9996	**0.0051**	**0.0121**
d__Bacteria;p__Firmicutes;c__Clostridia;o__Clostridia_UCG-014;f__Clostridia_UCG-014;g__Clostridia_UCG-014	0.08 (0.7)	0 (0)	0 (0)	1.08 (2.81)	**1.74 (1.99)**	**0.05 (0.1)**	**< 0.0001**	0.2785	**0.0003**	**0.0051**	0.0677	**0.0138**	0.9996	**0.022**
d__Bacteria;p__Firmicutes;c__Clostridia;o__Clostridia_vadinBB60_group;f__Clostridia_vadinBB60_group;g__Clostridia_vadinBB60_group	0.04 (0.12)	0 (0.04)	0 (0.02)	0.01 (0.04)	0.01 (0.04)	0.01 (0.02)	0.021	NT						
d__Bacteria;p__Firmicutes;c__Clostridia;o__Clostridiales;f__Clostridiaceae;__	0.1 (0.45)	0 (0)	0 (0)	0.04 (0.17)	0 (0)	0 (0)	**< 0.0001**	0.9776	0.9424	1	0.092	**0.0138**	**0.0138**	**0.0138**
d__Bacteria;p__Firmicutes;c__Clostridia;o__Clostridiales;f__Clostridiaceae;g__Candidatus_Arthromitus	0.03 (0.07)	0 (0)	0 (0)	0 (0.06)	0 (0.01)	**0.03 (0.01)**	**< 0.0001**	0.8294	0.9493	**0.0017**	**0.0358**	**0.0051**	0.7506	0.7721
d__Bacteria;p__Firmicutes;c__Clostridia;o__Lachnospirales;__;__	0.06 (0.08)	0.03 (0.13)	0 (0.02)	0.04 (0.07)	0.01 (0.05)	0 (0.05)	0.0146	NT						
d__Bacteria;p__Firmicutes;c__Clostridia;o__Lachnospirales;f__Lachnospiraceae;__	10.61 (5.36)	12.5 (8.16)	10.52 (6.18)	7.58 (4.47)	9.08 (3.3)	8.28 (6.1)	0.1012	NT						
d__Bacteria;p__Firmicutes;c__Clostridia;o__Lachnospirales;f__Lachnospiraceae;g__[Acetivibrio]_ethanolgignens_group	0.06 (0.19)	0 (0.04)	0 (0.07)	**0 (0)**	0 (0)	0 (0)	**< 0.0001**	**0.0138**	0.1567	0.1567	0.3578	0.5926	1	1
d__Bacteria;p__Firmicutes;c__Clostridia;o__Lachnospirales;f__Lachnospiraceae;g__A2	1.26 (1.7)	1.61 (1.64)	0.68 (0.9)	1.7 (1.27)	1.68 (1.55)	0.57 (0.49)	0.0369	NT						
d__Bacteria;p__Firmicutes;c__Clostridia;o__Lachnospirales;f__Lachnospiraceae;g__Acetatifactor	1.42 (0.94)	1.3 (0.67)	0.59 (0.61)	0.83 (0.94)	0.25 (0.21)	0.16 (0.17)	**< 0.0001**	0.8708	0.0574	0.1049	0.9906	0.0672	0.1049	**0.0294**
d__Bacteria;p__Firmicutes;c__Clostridia;o__Lachnospirales;f__Lachnospiraceae;g__Blautia	5.57 (3.66)	5.15 (3.03)	2.95 (2.58)	2.55 (3.33)	2.54 (2.35)	3.39 (6.72)	0.0247	NT						
d__Bacteria;p__Firmicutes;c__Clostridia;o__Lachnospirales;f__Lachnospiraceae;g__Dorea	0 (0.05)	0 (0.1)	0 (0)	0.03 (0.17)	0 (0)	0 (0)	0.0039	NT						
d__Bacteria;p__Firmicutes;c__Clostridia;o__Lachnospirales;f__Lachnospiraceae;g__GCA-900066575	1.04 (0.82)	0.94 (0.53)	0.57 (0.45)	0.63 (0.45)	0.43 (0.36)	**0.09 (0.21)**	**< 0.0001**	0.4913	0.0908	**0.0078**	0.9981	0.2833	0.6067	**0.0022**
d__Bacteria;p__Firmicutes;c__Clostridia;o__Lachnospirales;f__Lachnospiraceae;g__Lachnoclostridium	0.64 (0.56)	0.48 (0.36)	0.46 (0.54)	**2.85 (3.38)**	2.56 (5.52)	1.94 (1.73)	**< 0.0001**	**0.0011**	0.0672	0.0783	0.8708	0.9941	0.9998	0.7183
d__Bacteria;p__Firmicutes;c__Clostridia;o__Lachnospirales;f__Lachnospiraceae;g__Lachnospiraceae_FCS020_group	0.05 (0.05)	0.45 (0.31)	0.8 (1.12)	0.09 (0.26)	0.08 (0.49)	**0.12 (0.3)**	**< 0.0001**	0.9953	0.5851	**0.0063**	0.062	**0.0007**	0.9918	0.9782
d__Bacteria;p__Firmicutes;c__Clostridia;o__Lachnospirales;f__Lachnospiraceae;g__Lachnospiraceae_UCG-004	0.15 (0.24)	0.33 (0.21)	0.52 (0.41)	0.18 (0.43)	0.22 (0.18)	0.31 (0.42)	**0.0007**	0.9858	0.6821	0.3811	0.2833	**0.0022**	1	0.7857
d__Bacteria;p__Firmicutes;c__Clostridia;o__Lachnospirales;f__Lachnospiraceae;g__Lachnospiraceae_UCG-006	1.61 (1.5)	1.13 (0.81)	0.64 (0.97)	2.56 (1.92)	1.18 (1.83)	0.84 (2.03)	0.2205	NT						
d__Bacteria;p__Firmicutes;c__Clostridia;o__Lachnospirales;f__Lachnospiraceae;g__Marvinbryantia	0.44 (0.33)	0.16 (0.31)	0.16 (0.19)	**0.12 (0.05)**	0.06 (0.06)	**0.06 (0.04)**	**< 0.0001**	**0.0014**	0.3469	**0.0116**	0.1575	**0.0294**	0.2543	0.2833
d__Bacteria;p__Firmicutes;c__Clostridia;o__Lachnospirales;f__Lachnospiraceae;g__Roseburia	1.13 (1.76)	0.14 (0.86)	0 (0)	0.37 (0.57)	0.65 (0.83)	0.06 (0.16)	**< 0.0001**	0.0908	0.7857	0.1952	0.0783	**0.0007**	0.9147	0.166
d__Bacteria;p__Firmicutes;c__Clostridia;o__Lachnospirales;f__Lachnospiraceae;g__Sellimonas	0 (0.19)	0 (0.08)	0.29 (0.46)	0.23 (1.39)	0 (0.15)	0 (0.09)	0.0284	NT						
d__Bacteria;p__Firmicutes;c__Clostridia;o__Lachnospirales;f__Lachnospiraceae;g__Tuzzerella	1.05 (0.8)	0.38 (0.34)	0.67 (1.27)	0.1 (0.9)	**0.06 (0.05)**	**0.05 (0.09)**	**< 0.0001**	0.3811	**0.0027**	**0.0343**	0.0908	0.9147	0.3142	0.3118
d__Bacteria;p__Firmicutes;c__Clostridia;o__Monoglobales;f__Monoglobaceae;g__Monoglobus	0.1 (0.08)	0.06 (0.18)	0.16 (0.25)	0.03 (0.05)	0 (0)	**0 (0)**	**< 0.0001**	0.1375	0.0568	**0.0016**	0.9904	0.2019	**0.0081**	**0.0276**
d__Bacteria;p__Firmicutes;c__Clostridia;o__Oscillospirales;__;__	0.66 (0.46)	0.4 (0.28)	0.48 (0.46)	**0.14 (0.24)**	**0.2 (0.14)**	**0.08 (0.09)**	**< 0.0001**	**0.0489**	**0.0489**	**0.0042**	0.9147	0.9326	0.9906	**0.0349**
d__Bacteria;p__Firmicutes;c__Clostridia;o__Oscillospirales;f__[Eubacterium]_coprostanoligenes_group;g__[Eubacterium]_coprostanoligenes_group	0.2 (0.55)	0 (0.1)	0.21 (1.04)	**1.09 (1.54)**	**2.18 (2.25)**	**1.23 (2.2)**	**< 0.0001**	**0.0411**	**0.0026**	**0.2194**	0.2302	1	0.7183	0.9941
d__Bacteria;p__Firmicutes;c__Clostridia;o__Oscillospirales;f__Oscillospiraceae;__	3.98 (2.07)	2.06 (2.11)	1.05 (1.45)	1.57 (2.55)	**0.36 (0.43)**	0.21 (0.28)	**< 0.0001**	0.1206	**0.0205**	0.2271	0.0783	**0.0011**	**0.0246**	**0.0042**
d__Bacteria;p__Firmicutes;c__Clostridia;o__Oscillospirales;f__Oscillospiraceae;g__Colidextribacter	5.24 (3.03)	5.95 (4.93)	2.74 (2.74)	3.55 (2.55)	3.2 (1.8)	**0.71 (0.79)**	**< 0.0001**	0.3469	0.1381	**0.0116**	1	**0.035**	0.7857	**0.0005**
d__Bacteria;p__Firmicutes;c__Clostridia;o__Oscillospirales;f__Oscillospiraceae;g__Intestinimonas	0.46 (0.3)	0.24 (0.18)	0.19 (0.12)	**0.15 (0.11)**	**0.06 (0.09)**	**0 (0.02)**	**< 0.0001**	**0.0095**	**0.0472**	**0.0005**	0.1047	**0.0489**	0.0521	**0.0028**
d__Bacteria;p__Firmicutes;c__Clostridia;o__Oscillospirales;f__Oscillospiraceae;g__NK4A214_group	0.04 (0.16)	0 (0)	0 (0)	0.18 (0.21)	0 (0)	0 (0)	**< 0.0001**	0.3549	0.7365	1	**0.034**	**0.034**	**0.0013**	**0.0005**
d__Bacteria;p__Firmicutes;c__Clostridia;o__Oscillospirales;f__Oscillospiraceae;g__Oscillibacter	1.89 (1.45)	1.64 (1.39)	0.94 (0.69)	2.03 (1.56)	2.26 (1.79)	0.85 (0.73)	**< 0.0001**	0.9906	0.5296	1	0.7529	0.0672	1	**0.0014**
d__Bacteria;p__Firmicutes;c__Clostridia;o__Oscillospirales;f__Oscillospiraceae;g__Oscillospira	0 (0.06)	0 (0)	0 (0)	0 (0.07)	0 (0)	0 (0)	0.0046	NT						
d__Bacteria;p__Firmicutes;c__Clostridia;o__Oscillospirales;f__Oscillospiraceae;g__UCG-003	0.02 (0.17)	0.06 (0.12)	0 (0.07)	0.1 (0.17)	0 (0.06)	0 (0)	0.005	NT						
d__Bacteria;p__Firmicutes;c__Clostridia;o__Oscillospirales;f__Oscillospiraceae;g__UCG-005	0 (0.03)	0 (0.02)	0 (0.03)	0.08 (0.15)	0.08 (0.18)	0 (0)	0.0012	NT						
d__Bacteria;p__Firmicutes;c__Clostridia;o__Oscillospirales;f__Oscillospiraceae;g__uncultured	0.86 (1.64)	0.84 (0.95)	0.66 (1.11)	1.3 (1.79)	1.04 (1.45)	0.33 (0.33)	0.0029	NT						
d__Bacteria;p__Firmicutes;c__Clostridia;o__Oscillospirales;f__Ruminococcaceae;__	1.14 (0.56)	0.68 (0.38)	0.28 (0.19)	**0.25 (0.5)**	**0.09 (0.13)**	**0.02 (0.06)**	**< 0.0001**	**0.035**	**0.0011**	**0.0111**	0.1787	**0.0017**	**0.0141**	**0.0017**
d__Bacteria;p__Firmicutes;c__Clostridia;o__Oscillospirales;f__Ruminococcaceae;g__Anaerotruncus	0.19 (0.11)	0.06 (0.04)	0.05 (0.07)	0.02 (0.05)	0 (0.04)	0 (0.03)	**< 0.0001**	0.0661	0.0717	0.1571	**0.0052**	**0.0063**	0.9175	0.7475
d__Bacteria;p__Firmicutes;c__Clostridia;o__Oscillospirales;f__Ruminococcaceae;g__Incertae_Sedis	1.2 (1.02)	0.26 (0.43)	0.16 (0.22)	0.42 (0.69)	0.15 (0.36)	0.11 (0.1)	**< 0.0001**	0.0908	0.8165	0.9239	**0.0022**	**0.0005**	0.2543	**0.0095**
d__Bacteria;p__Firmicutes;c__Clostridia;o__Oscillospirales;f__Ruminococcaceae;g__Negativibacillus	0.13 (0.2)	0 (0.06)	0 (0.06)	**0 (0)**	0 (0)	0 (0)	**< 0.0001**	**0.0003**	0.5871	0.4517	**0.0011**	**0.0009**	1	0.9976
d__Bacteria;p__Firmicutes;c__Clostridia;o__Oscillospirales;f__Ruminococcaceae;g__UBA1819	0 (0.02)	0 (0)	0 (0)	0.02 (0.06)	0.03 (0.05)	0 (0.02)	0.0015	NT						
d__Bacteria;p__Firmicutes;c__Clostridia;o__Oscillospirales;f__Ruminococcaceae;g__uncultured	0.3 (0.21)	0.31 (0.46)	0.35 (0.33)	0.03 (0.09)	0.05 (0.1)	0.02 (0.05)	**< 0.0001**	**0.017**	0.12	**0.0005**	0.9965	0.6067	0.9144	0.8648
d__Bacteria;p__Firmicutes;c__Clostridia;o__Oscillospirales;f__UCG-010;g__UCG-010	0.4 (0.44)	0.1 (0.51)	0 (0.03)	0.33 (0.56)	0 (0.1)	0 (0.01)	**< 0.0001**	0.9941	0.323	0.9749	0.2533	**0.0005**	**0.0085**	**0.0017**
d__Bacteria;p__Firmicutes;c__Clostridia;o__Peptococcales;f__Peptococcaceae;g__uncultured	6.37 (2.88)	2.78 (4.15)	1.58 (2.66)	3.37 (3.03)	2.17 (3.17)	0.4 (2.03)	**< 0.0001**	**0.035**	0.9906	0.7181	0.1381	**0.0014**	0.9147	0.1568
d__Bacteria;p__Firmicutes;c__Clostridia;o__Peptostreptococcales-Tissierellales;__;__	1.38 (1.74)	0.61 (0.85)	0.19 (0.51)	0.01 (0.17)	0 (0)	0 (0)	**< 0.0001**	**0.0007**	**0.0001**	**0.0052**	0.4913	**0.0413**	0.0763	0.4071
d__Bacteria;p__Firmicutes;c__Clostridia;o__Peptostreptococcales-Tissierellales;f__Anaerovoracaceae;g__[Eubacterium]_brachy_group	0.05 (0.07)	0.03 (0.03)	0.03 (0.04)	0.08 (0.18)	0.02 (0.07)	0.01 (0.02)	0.0167	NT						
d__Bacteria;p__Firmicutes;c__Clostridia;o__Peptostreptococcales-Tissierellales;f__Anaerovoracaceae;g__Family_XIII_AD3011_group	0.17 (0.17)	0.05 (0.13)	0 (0.07)	0 (0)	0 (0)	0 (0)	**< 0.0001**	**0.0005**	**0.0005**	0.1567	0.3636	**0.0385**	1	1
d__Bacteria;p__Firmicutes;c__Clostridia;o__Peptostreptococcales-Tissierellales;f__Peptostreptococcaceae;__	3.2 (1.73)	0.95 (0.98)	0.81 (2.27)	0 (0)	0 (0)	0 (0)	**< 0.0001**	**0.0001**	**0.0005**	**0.0138**	**0.0078**	**0.048**	1	1
d__Bacteria;p__Firmicutes;c__Clostridia;o__Peptostreptococcales-Tissierellales;f__Peptostreptococcaceae;g__uncultured	3.3 (1.64)	0.94 (1.07)	0.87 (2.43)	0 (0)	0 (0)	0 (0)	**< 0.0001**	**0.0001**	**0.0009**	**0.0138**	**0.0141**	**0.0407**	0.9424	1
d__Bacteria;p__Proteobacteria;c__Gammaproteobacteria;o__Enterobacterales;__;__	0.21 (0.55)	0.38 (1.33)	0.34 (0.97)	0.05 (0.32)	0.03 (0.09)	0 (0.03)	**0.0007**	0.9793	0.1881	**0.027**	0.9323	0.9998	0.7999	**0.0225**
d__Bacteria;p__Verrucomicrobiota;c__Verrucomicrobiae;o__Verrucomicrobiales;f__Akkermansiaceae;g__Akkermansia	0 (0.01)	0 (0.01)	0 (0.01)	17.32 (19.9)	0 (0)	21.17 (18.52)	**< 0.0001**	**0.0318**	0.953	**0.0068**	1	1	**0.0226**	**0.6813**

All data (n = 12 per group) are expressed as the median and interquartile range (IQR). Only genera that are detected in more than 10 % samples were selected and processed for comparizons among the groups. When a p value was less than 0.000714 in the KW test comparing the six groups, the Steel-Dwass test was applied to evaluate differences in the relative abundance between the indicated two groups.The p value of 0.000714 in the KW test was calculated using the Bonferroni correction based on the total number of tests (70, p = 0.05/70 = 0.000714). Bold figures indicate a significant difference between the indicated two groups (p < 0.05). G1, G2, and G3 show the 1st, 2nd, and 3rd generation, respectively. KW, Kruskal-Wallis test; CON, a group fed control diets; KES, a group fed kestose-supplemented diets.
