# Supplementary material for: Dietary supplementation with 1‐kestose induces altered locomotor activity and increased striatal dopamine levels with a change in gut microbiota in male mice
Source: Physiol Rep. 2023 Dec 6;11(23):e15882. doi: 10.14814/phy2.15882 (PMC10698829; doi:10.14814/phy2.15882)
Supplement: Supplementary file 3 — Table S3. [file PHY2-11-e15882-s004.pdf]

Supplementary table 3. Relative abundance at species levels

	CON			KES										
	G1	G2	G3	G1	G2	G3								
		Median (IQR)			Median (IQR)		KW	G1 (CON vs KES)	G2 (CON vs KES)	G3 (CON vs KES)	CON (G2 vs G1)	CON (G3 vs G1)	KES (G2 vs G1)	KES (G3 vs G1)
d__Bacteria;p__Actinobacteriota;c__Actinobacteria;o__Bifidobacteriales;f__Bifidobacteriaceae;g__Bifidobacterium;__	0 (0)	0 (0.01)	4.05 (3.45)	0 (0.02)	10.16 (10.8)	8.59 (3.78)	**< .0001**	0.8067	**0.0023**	0.1205	0.5014	**0.0032**	**0.0004**	**0.0004**
d__Bacteria;p__Actinobacteriota;c__Coriobacteriia;o__Coriobacteriales;__;__;__	0.03 (0.07)	0.01 (0.05)	0 (0.07)	0.02 (0.05)	0.02 (0.02)	0 (0.02)	0.5795	NT						
d__Bacteria;p__Actinobacteriota;c__Coriobacteriia;o__Coriobacteriales;f__Eggerthellaceae;__;__	0.74 (1.08)	1.63 (1.5)	0.92 (0.93)	0.59 (0.41)	0.17 (0.22)	0.3 (0.33)	**< .0001**	0.9858	**0.0009**	**0.0034**	0.2271	0.7857	0.0141	0.3511
d__Bacteria;p__Actinobacteriota;c__Coriobacteriia;o__Coriobacteriales;f__Eggerthellaceae;g__Enterorhabdus;s__mouse_gut	0 (0)	0 (0)	0 (0.13)	0 (0)	0 (0)	0.07 (0.13)	**< .0001**	1	0.9424	0.46	1	0.9424	0.9424	**0.0005**
d__Bacteria;p__Actinobacteriota;c__Coriobacteriia;o__Coriobacteriales;f__Eggerthellaceae;g__uncultured;s__uncultured_Coriobacteriales	0 (0)	0 (0.05)	0.02 (0.04)	0 (0)	0 (0)	0 (0)	0.0015	NT						
d__Bacteria;p__Bacteroidota;c__Bacteroidia;o__Bacteroidales;f__Bacteroidaceae;g__Bacteroides;s__Bacteroides_caccae	7.73 (3.03)	4.53 (3.52)	0.01 (2.99)	8.07 (5.18)	10.01 (6.71)	9.36 (12.09)	**0.0004**	0.998	0.1474	0.373	**0.0294**	**0.0058**	0.9941	1
d__Bacteria;p__Bacteroidota;c__Bacteroidia;o__Bacteroidales;f__Muribaculaceae;g__Muribaculaceae;s__uncultured_bacterium	0 (0)	0 (0.03)	5.7 (8.55)	8.21 (4.77)	1.43 (3.46)	2.71 (3.97)	**< .0001**	**0.0001**	0.1365	0.9998	0.4934	**0.034**	**0.0116**	**0.0205**
d__Bacteria;p__Bacteroidota;c__Bacteroidia;o__Bacteroidales;f__Tannerellaceae;__;__	8.31 (4.31)	12.83 (15.96)	9.06 (3.28)	8.21 (4.44)	13.01 (5.72)	6.44 (8.05)	0.0235	NT						
d__Bacteria;p__Deferribacterota;c__Deferribacteres;o__Deferribacterales;f__Deferribacteraceae;g__Mucispirillum;s__Mucispirillum_schaedleri	0.54 (1.87)	0 (0.92)	0 (0)	0.01 (0.24)	0 (0)	0 (0)	**< .0001**	0.1287	0.4934	1	0.1792	**0.0005**	0.0763	0.0763
d__Bacteria;p__Firmicutes;c__Bacilli;o__Erysipelotrichales;f__Erysipelatoclostridiaceae;g__Candidatus_Stoquefichus;s__unidentified	0.16 (0.28)	0 (0)	0 (0)	0.06 (0.1)	0 (0)	0 (0)	**< .0001**	0.3054	1	1	**0.0017**	**0.0017**	**0.0138**	**0.0138**
d__Bacteria;p__Firmicutes;c__Bacilli;o__Erysipelotrichales;f__Erysipelatoclostridiaceae;g__Erysipelatoclostridium;__	0 (0)	0 (0)	0 (0)	0.6 (3.57)	0 (0)	0 (1.15)	0.0105	NT						
d__Bacteria;p__Firmicutes;c__Bacilli;o__Erysipelotrichales;f__Erysipelatoclostridiaceae;g__Erysipelatoclostridium;s__[Clostridium]_cocleatum	6.82 (4.1)	6.84 (5.4)	6.93 (4.56)	0.98 (3.96)	1.56 (1.53)	1.2 (3.48)	**< .0001**	0.0555	**0.035**	**0.0077**	0.9998	0.9858	0.9964	0.9999
d__Bacteria;p__Firmicutes;c__Bacilli;o__Erysipelotrichales;f__Erysipelatoclostridiaceae;g__Erysipelatoclostridium;s__Massiliomicrobiota_timonensis	0.11 (0.16)	0.04 (0.1)	0.07 (0.09)	0 (0.05)	0.09 (0.16)	0 (0.01)	0.003	NT						
d__Bacteria;p__Firmicutes;c__Bacilli;o__Erysipelotrichales;f__Erysipelotrichaceae;__;__	0.08 (0.15)	0 (0)	0 (0)	0.07 (0.09)	0 (0.05)	0 (0)	**< .0001**	0.998	0.8483	0.7365	**0.0186**	**0.0051**	0.273	**0.0052**
d__Bacteria;p__Firmicutes;c__Bacilli;o__Erysipelotrichales;f__Erysipelotrichaceae;g__Erysipelotrichaceae;s__uncultured_bacterium	0.06 (0.19)	0 (0)	0 (0)	0.03 (0.09)	0 (0)	0 (0)	**< .0001**	0.9996	0.9939	1	0.065	**0.0138**	**0.0226**	**0.0017**
d__Bacteria;p__Firmicutes;c__Bacilli;o__Erysipelotrichales;f__Erysipelotrichaceae;g__Faecalibaculum;s__Faecalibaculum_rodentium	0 (0)	17.6 (21.94)	23.8 (10)	0 (0)	20.13 (9.1)	25.17 (8.39)	**< .0001**	0.9424	0.9998	0.9998	**0.0001**	**0.0005**	**0.0002**	**0.0002**
d__Bacteria;p__Firmicutes;c__Bacilli;o__Erysipelotrichales;f__Erysipelotrichaceae;g__Turicibacter;__	0.18 (0.69)	0.09 (0.25)	0.24 (1.78)	3.37 (2.47)	0.14 (0.46)	0 (0)	**< .0001**	**0.022**	1	**0.0005**	0.9855	0.9826	**0.0074**	**0.0005**
d__Bacteria;p__Firmicutes;c__Bacilli;o__Erysipelotrichales;f__Erysipelotrichaceae;g__uncultured;s__unidentified	0 (0)	0 (0)	0 (0)	0 (0.1)	0.05 (0.09)	0 (0.01)	**< .0001**	0.2766	**0.0005**	0.4934	0.9424	0.9424	0.8379	0.7105
d__Bacteria;p__Firmicutes;c__Bacilli;o__Lactobacillales;f__Enterococcaceae;g__Enterococcus;__	0.12 (0.6)	0.06 (0.24)	0 (0.04)	0.04 (0.06)	0 (0)	0 (0)	**< .0001**	0.6615	**0.0276**	0.6617	0.9323	**0.0328**	0.062	**0.0104**
d__Bacteria;p__Firmicutes;c__Bacilli;o__Lactobacillales;f__Lactobacillaceae;g__Lactobacillus;__	0.87 (1.05)	0.08 (0.4)	0 (0.31)	1.58 (2.1)	0.11 (0.39)	0 (0)	**< .0001**	0.4168	0.9746	0.8758	0.1175	**0.0044**	**0.0116**	**0.0006**
d__Bacteria;p__Firmicutes;c__Bacilli;o__Lactobacillales;f__Streptococcaceae;g__Lactococcus;__	1 (0.93)	0.79 (0.43)	0.72 (0.79)	0.33 (0.31)	0.15 (0.09)	0.13 (0.3)	**< .0001**	0.1575	**0.0007**	**0.0116**	0.9981	0.9958	0.4913	0.2543
d__Bacteria;p__Firmicutes;c__Bacilli;o__Lactobacillales;f__Streptococcaceae;g__Streptococcus;__	0.02 (0.05)	0 (0)	0 (0)	0 (0.01)	0 (0.03)	0 (0)	0.0163	NT						
d__Bacteria;p__Firmicutes;c__Bacilli;o__Lactobacillales;f__Streptococcaceae;g__Streptococcus;s__Streptococcus_danieliae	0 (0)	0 (0)	0.05 (0.06)	0 (0)	0 (0)	0.01 (0.02)	**< .0001**	0.9424	0.9424	0.3093	1	**0.0051**	0.9424	0.237
d__Bacteria;p__Firmicutes;c__Bacilli;o__RF39;f__RF39;g__RF39;__	0 (0)	0 (0)	0 (0)	0 (0.04)	0 (0.02)	0 (0.02)	0.0387	NT						
d__Bacteria;p__Firmicutes;c__Bacilli;o__Staphylococcales;f__Staphylococcaceae;g__Staphylococcus;s__Staphylococcus_carnosus	0.02 (0.05)	0.01 (0.03)	0 (0.03)	0.02 (0.02)	0 (0)	0 (0)	0.0421	NT						
d__Bacteria;p__Firmicutes;c__Clostridia;__;__;__;__	0.88 (0.9)	0.33 (0.94)	0.44 (0.65)	0.17 (0.21)	0.07 (0.06)	0 (0.07)	**< .0001**	**0.0064**	**0.0009**	**0.0028**	0.7529	0.3811	**0.0489**	**0.0082**
d__Bacteria;p__Firmicutes;c__Clostridia;o__Christensenellales;f__Christensenellaceae;__;__	0.03 (0.02)	0.01 (0.03)	0.04 (0.03)	0 (0.01)	0 (0.01)	0 (0)	**< .0001**	0.0539	0.6251	**0.0005**	0.3949	1	1	0.4934
d__Bacteria;p__Firmicutes;c__Clostridia;o__Christensenellales;f__Christensenellaceae;g__Christensenellaceae_R-7_group;__	0 (0.18)	0.19 (0.25)	0.11 (0.15)	0 (0)	0 (0)	0 (0)	**< .0001**	0.8577	**0.0017**	**0.0124**	0.2591	0.3944	0.9424	0.9939
d__Bacteria;p__Firmicutes;c__Clostridia;o__Christensenellales;f__Christensenellaceae;g__Christensenellaceae_R-7_group;s__unidentified	0.05 (0.06)	0 (0)	0 (0)	0.04 (0.12)	0 (0)	0 (0)	**< .0001**	0.9997	0.7365	1	**0.0051**	**0.0051**	**0.0064**	**0.0005**
d__Bacteria;p__Firmicutes;c__Clostridia;o__Clostridia_UCG-014;f__Clostridia_UCG-014;g__Clostridia_UCG-014;__	0 (0)	0 (0)	0 (0)	0.04 (0.8)	0.22 (1.69)	0 (0)	**< .0001**	**0.034**	**0.0005**	0.9424	1	1	0.6179	0.0938
d__Bacteria;p__Firmicutes;c__Clostridia;o__Clostridia_UCG-014;f__Clostridia_UCG-014;g__Clostridia_UCG-014;s__uncultured_Acetivibrio	0 (0.02)	0 (0)	0 (0)	0.01 (0.44)	0.08 (0.18)	0 (0.06)	**< .0001**	0.7105	**0.0014**	0.1567	**0.0226**	0.4934	0.7074	0.9581
d__Bacteria;p__Firmicutes;c__Clostridia;o__Clostridia_UCG-014;f__Clostridia_UCG-014;g__Clostridia_UCG-014;s__uncultured_Lactococcus	0.01 (0.67)	0 (0)	0 (0)	0.63 (2.27)	0.02 (0.97)	0 (0.06)	**< .0001**	0.4022	0.1796	0.1567	0.1796	0.0763	0.4022	**0.0225**
d__Bacteria;p__Firmicutes;c__Clostridia;o__Clostridia_vadinBB60_group;f__Clostridia_vadinBB60_group;g__Clostridia_vadinBB60_group;__	0.02 (0.08)	0 (0.04)	0 (0.02)	0.01 (0.04)	0 (0.03)	0 (0.01)	0.3847	NT						
d__Bacteria;p__Firmicutes;c__Clostridia;o__Clostridia_vadinBB60_group;f__Clostridia_vadinBB60_group;g__Clostridia_vadinBB60_group;s__uncultured_Clostridia	0 (0.04)	0 (0)	0 (0)	0 (0)	0 (0.01)	0 (0)	0.1161	NT						
d__Bacteria;p__Firmicutes;c__Clostridia;o__Clostridiales;f__Clostridiaceae;__;__	0.1 (0.45)	0 (0)	0 (0)	0.04 (0.17)	0 (0)	0 (0)	**< .0001**	0.9776	0.9424	1	0.092	**0.0138**	**0.0138**	**0.0138**
d__Bacteria;p__Firmicutes;c__Clostridia;o__Clostridiales;f__Clostridiaceae;g__Candidatus_Arthromitus;s__Candidatus_Arthromitus	0.03 (0.07)	0 (0)	0 (0)	0 (0.06)	0 (0.01)	0.03 (0.01)	**< .0001**	0.8294	0.9493	**0.0017**	**0.0358**	**0.0051**	0.7506	0.7721
d__Bacteria;p__Firmicutes;c__Clostridia;o__Lachnospirales;__;__;__	0.06 (0.08)	0.03 (0.13)	0 (0.02)	0.04 (0.07)	0.01 (0.05)	0 (0.05)	0.0146	NT						
d__Bacteria;p__Firmicutes;c__Clostridia;o__Lachnospirales;f__Lachnospiraceae;__;__	10.61 (5.36)	12.5 (8.16)	10.52 (6.18)	7.58 (4.47)	9.08 (3.3)	8.28 (6.1)	0.1012	NT						
d__Bacteria;p__Firmicutes;c__Clostridia;o__Lachnospirales;f__Lachnospiraceae;g__[Acetivibrio]_ethanolgignens_group;s__uncultured_bacterium	0.06 (0.19)	0 (0.04)	0 (0.07)	0 (0)	0 (0)	0 (0)	**< .0001**	**0.0138**	0.1567	0.1567	0.3578	0.5926	1	1
d__Bacteria;p__Firmicutes;c__Clostridia;o__Lachnospirales;f__Lachnospiraceae;g__A2;__	0 (0)	0 (0)	0 (0)	0.06 (1.42)	0.79 (2.09)	0.36 (0.73)	**< .0001**	0.1112	0.0186	0.0051	0.9976	0.9424	0.8483	0.9993
d__Bacteria;p__Firmicutes;c__Clostridia;o__Lachnospirales;f__Lachnospiraceae;g__Acetatifactor;__	0.43 (0.28)	0.83 (0.33)	0.21 (0.28)	0.11 (0.15)	0.04 (0.1)	0.01 (0.05)	**< .0001**	0.0661	**0.0056**	0.08	**0.0347**	0.7512	0.8137	0.1423
d__Bacteria;p__Firmicutes;c__Clostridia;o__Lachnospirales;f__Lachnospiraceae;g__Acetatifactor;s__Acetatifactor_muris	1.08 (0.96)	0.39 (0.32)	0.41 (0.27)	0.7 (1.13)	0.19 (0.21)	0.16 (0.18)	**< .0001**	0.9941	0.5296	0.1381	0.2019	0.0672	0.0908	**0.0414**
d__Bacteria;p__Firmicutes;c__Clostridia;o__Lachnospirales;f__Lachnospiraceae;g__Blautia;__	0.02 (0.08)	0.11 (0.13)	0.12 (0.16)	0 (0)	0 (0)	0 (0)	**< .0001**	0.0763	**0.0051**	**0.0005**	0.46	0.0717	1	1
d__Bacteria;p__Firmicutes;c__Clostridia;o__Lachnospirales;f__Lachnospiraceae;g__Blautia;s__Lachnospiraceae_bacterium	1.12 (1.1)	1.51 (1.14)	1.21 (0.77)	0.88 (3.04)	2.41 (1.57)	3.24 (6.76)	0.0857	NT						
d__Bacteria;p__Firmicutes;c__Clostridia;o__Lachnospirales;f__Lachnospiraceae;g__Blautia;s__uncultured_Clostridiales							**< .0001**	**0.0013**	**0.0104**	**0.0004**	0.6067	0.2019	0.9926	0.0946
d__Bacteria;p__Firmicutes;c__Clostridia;o__Lachnospirales;f__Lachnospiraceae;g__Coprococcus;s__Clostridium_sp.	0 (0)	0 (0.04)	0 (0)	0 (0)	0 (0)	0 (0)	0.0021	NT						
d__Bacteria;p__Firmicutes;c__Clostridia;o__Lachnospirales;f__Lachnospiraceae;g__Dorea;__	0 (0.05)	0 (0.1)	0 (0)	0.03 (0.17)	0 (0)	0 (0)	0.0039	NT						
d__Bacteria;p__Firmicutes;c__Clostridia;o__Lachnospirales;f__Lachnospiraceae;g__GCA-900066575;s__uncultured_Clostridium	1.04 (0.82)	0.94 (0.53)	0.57 (0.45)	0.63 (0.45)	0.43 (0.36)	0.09 (0.21)	**< .0001**	0.4913	0.0908	**0.0078**	0.9981	0.2833	0.6067	**0.0022**
d__Bacteria;p__Firmicutes;c__Clostridia;o__Lachnospirales;f__Lachnospiraceae;g__Lachnoclostridium;__	0.64 (0.6)	0.47 (0.42)	0.46 (0.42)	2.81 (3.38)	2.1 (5.53)	1.82 (2.18)	**< .0001**	**0.0022**	0.1787	0.5296	0.8165	0.9606	0.9941	0.6067
d__Bacteria;p__Firmicutes;c__Clostridia;o__Lachnospirales;f__Lachnospiraceae;g__Lachnoclostridium;s__Clostridium_fusiformis	0 (0)	0 (0)	0 (0)	0 (0)	0 (0.34)	0 (0.24)	0.0216	NT						
d__Bacteria;p__Firmicutes;c__Clostridia;o__Lachnospirales;f__Lachnospiraceae;g__Lachnospiraceae_FCS020_group;s__mouse_gut	0 (0)	0.44 (0.36)	0.78 (1.16)	0 (0.21)	0.07 (0.48)	0.09 (0.31)	**< .0001**	0.7658	0.5015	0.006	0.0026	0.0004	0.955	0.9401
d__Bacteria;p__Firmicutes;c__Clostridia;o__Lachnospirales;f__Lachnospiraceae;g__Lachnospiraceae_FCS020_group;s__uncultured_prokaryote	0.04 (0.06)	0.01 (0.05)	0.01 (0.09)	0 (0.04)	0 (0.02)	0 (0.04)	0.1945	NT						
d__Bacteria;p__Firmicutes;c__Clostridia;o__Lachnospirales;f__Lachnospiraceae;g__Lachnospiraceae_NK4A136_group;s__Trichinella_pseudospiralis	0 (0)	0 (0)	0 (0)	0 (0)	0 (0.03)	0 (0.02)	0.0945	NT						
d__Bacteria;p__Firmicutes;c__Clostridia;o__Lachnospirales;f__Lachnospiraceae;g__Lachnospiraceae_UCG-004;__	0.15 (0.24)	0.33 (0.21)	0.52 (0.41)	0.18 (0.43)	0.22 (0.18)	0.31 (0.42)	0.0007	NT						
d__Bacteria;p__Firmicutes;c__Clostridia;o__Lachnospirales;f__Lachnospiraceae;g__Lachnospiraceae_UCG-006;s__Clostridium_sp.	1.61 (1.5)	1.13 (0.81)	0.64 (0.97)	2.56 (1.92)	1.18 (1.83)	0.84 (2.03)	0.2205	NT						
d__Bacteria;p__Firmicutes;c__Clostridia;o__Lachnospirales;f__Lachnospiraceae;g__Marvinbryantia;s__Clostridiales_bacterium	0.44 (0.33)	0.16 (0.31)	0.16 (0.19)	0.12 (0.05)	0.06 (0.06)	0.06 (0.04)	**< .0001**	**0.0014**	0.3469	**0.0116**	0.1575	**0.0294**	0.2543	0.2833
d__Bacteria;p__Firmicutes;c__Clostridia;o__Lachnospirales;f__Lachnospiraceae;g__Roseburia;__	1.11 (1.47)	0.13 (0.78)	0 (0)	0.02 (0.46)	0.04 (0.34)	0 (0.02)	**< .0001**	**0.0058**	0.7768	1	0.1787	**0.0007**	1	0.6251
d__Bacteria;p__Firmicutes;c__Clostridia;o__Lachnospirales;f__Lachnospiraceae;g__Roseburia;s__mouse_gut	0 (0.04)	0 (0.03)	0 (0)	0.17 (0.41)	0.31 (0.67)	0.05 (0.08)	**< .0001**	0.1371	**0.0079**	**0.0138**	0.9629	0.1567	0.8687	0.8648
d__Bacteria;p__Firmicutes;c__Clostridia;o__Lachnospirales;f__Lachnospiraceae;g__Sellimonas;s__uncultured_bacterium	0 (0.19)	0 (0.08)	0.24 (0.46)	0.23 (1.39)	0 (0.15)	0 (0.09)	0.0246	NT						
d__Bacteria;p__Firmicutes;c__Clostridia;o__Lachnospirales;f__Lachnospiraceae;g__Tuzzerella;s__uncultured_organism	1.05 (0.8)	0.38 (0.34)	0.67 (1.27)	0.1 (0.9)	0.06 (0.05)	0.05 (0.09)	**< .0001**	0.3811	**0.0027**	**0.0343**	0.0908	0.9147	0.3142	0.3118
d__Bacteria;p__Firmicutes;c__Clostridia;o__Lachnospirales;f__Lachnospiraceae;g__UC5-1-2E3;__	0 (0)	0.02 (0.05)	0.02 (0.05)	0 (0)	0 (0)	0 (0)	0.0004	NT						
d__Bacteria;p__Firmicutes;c__Clostridia;o__Monoglobales;f__Monoglobaceae;g__Monoglobus;__	0.03 (0.09)	0.06 (0.15)	0.16 (0.24)	0.02 (0.04)	0 (0)	0 (0)	**< .0001**	0.996	0.0786	**0.0016**	0.9992	**0.0196**	**0.0449**	0.1272
d__Bacteria;p__Firmicutes;c__Clostridia;o__Monoglobales;f__Monoglobaceae;g__Monoglobus;s__unidentified	0.05 (0.05)	0.01 (0.04)	0.01 (0.03)	0 (0.04)	0 (0)	0 (0)	**< .0001**	0.0863	0.1796	0.0763	0.1678	0.0717	0.3577	0.1567
d__Bacteria;p__Firmicutes;c__Clostridia;o__Oscillospirales;__;__;__	0.66 (0.46)	0.4 (0.28)	0.48 (0.46)	0.14 (0.24)	0.2 (0.14)	0.08 (0.09)	**< .0001**	**0.0489**	**0.0489**	**0.0042**	0.9147	0.9326	0.9906	**0.0349**
d__Bacteria;p__Firmicutes;c__Clostridia;o__Oscillospirales;f__[Eubacterium]_coprostanoligenes_group;g__[Eubacterium]_coprostanoligenes_group;__	0.2 (0.55)	0 (0.1)	0.21 (1.04)	1.09 (1.54)	2.18 (2.25)	1.23 (2.2)	**< .0001**	**0.0411**	**0.0026**	0.2194	0.2302	1	0.7183	0.9941
d__Bacteria;p__Firmicutes;c__Clostridia;o__Oscillospirales;f__Oscillospiraceae;__;__	3.98 (2.07)	2.06 (2.11)	1.05 (1.45)	1.57 (2.55)	0.36 (0.43)	0.21 (0.28)	**< .0001**	0.1206	**0.0205**	0.2271	0.0783	**0.0011**	**0.0246**	**0.0042**
d__Bacteria;p__Firmicutes;c__Clostridia;o__Oscillospirales;f__Oscillospiraceae;g__Colidextribacter;__	0.72 (1.08)	0.82 (1.11)	0.29 (0.46)	1.45 (1.1)	1.98 (2.15)	0.47 (0.53)	**< .0001**	0.4168	0.2543	0.9996	0.9996	0.0783	0.8449	**0.0052**
d__Bacteria;p__Firmicutes;c__Clostridia;o__Oscillospirales;f__Oscillospiraceae;g__Colidextribacter;s__bacterium_enrichment	0 (0.06)	0 (0.05)	0 (0)	0 (0.02)	0 (0.06)	0 (0.03)	0.6605	NT						
d__Bacteria;p__Firmicutes;c__Clostridia;o__Oscillospirales;f__Oscillospiraceae;g__Colidextribacter;s__Firmicutes_bacterium	4.54 (2.9)	5.33 (5.55)	2.58 (2.05)	2.25 (1.38)	0.93 (0.79)	0.26 (0.42)	**< .0001**	0.0574	**0.0116**	**0.0017**	**0.9998**	**0.0489**	**0.0205**	**0.0021**
d__Bacteria;p__Firmicutes;c__Clostridia;o__Oscillospirales;f__Oscillospiraceae;g__Intestinimonas;__	0.46 (0.3)	0.24 (0.18)	0.19 (0.12)	0.15 (0.11)	0.06 (0.09)	0 (0.02)	**< .0001**	**0.0095**	**0.0472**	**0.0005**	0.1047	**0.0489**	0.0521	**0.0028**
d__Bacteria;p__Firmicutes;c__Clostridia;o__Oscillospirales;f__Oscillospiraceae;g__NK4A214_group;s__unidentified	0.04 (0.16)	0 (0)	0 (0)	0.18 (0.21)	0 (0)	0 (0)	**< .0001**	0.3549	0.7365	1	**0.034**	**0.034**	**0.0013**	**0.0005**
d__Bacteria;p__Firmicutes;c__Clostridia;o__Oscillospirales;f__Oscillospiraceae;g__Oscillibacter;__	0.09 (0.14)	0.01 (0.05)	0 (0)	0.09 (0.15)	0 (0.03)	0 (0)	**< .0001**	0.9998	0.9084	0.9868	0.1624	**0.0124**	0.0539	**0.0051**
d__Bacteria;p__Firmicutes;c__Clostridia;o__Oscillospirales;f__Oscillospiraceae;g__Oscillibacter;s__[Clostridium]_leptum	1.04 (1.27)	0.44 (0.65)	0.55 (0.59)	1.52 (1.16)	1.71 (1.77)	0.57 (0.49)	**< .0001**	0.9794	**0.0141**	0.999	0.1575	0.0783	0.9479	**0.0414**
d__Bacteria;p__Firmicutes;c__Clostridia;o__Oscillospirales;f__Oscillospiraceae;g__Oscillibacter;s__Clostridiales_bacterium	0 (0.04)	0.02 (0.07)	0.03 (0.1)	0 (0.03)	0 (0.04)	0 (0)	0.0388	NT						
d__Bacteria;p__Firmicutes;c__Clostridia;o__Oscillospirales;f__Oscillospiraceae;g__Oscillibacter;s__gut_metagenome	0 (0)	0 (0)	0 (0)	0 (0.08)	0 (0.04)	0 (0)	0.0086	NT						
d__Bacteria;p__Firmicutes;c__Clostridia;o__Oscillospirales;f__Oscillospiraceae;g__Oscillibacter;s__Oscillibacter_sp.	0.5 (0.57)	0.75 (0.87)	0.24 (0.3)	0.61 (0.44)	0.18 (0.36)	0.17 (0.31)	0.0023	NT						
d__Bacteria;p__Firmicutes;c__Clostridia;o__Oscillospirales;f__Oscillospiraceae;g__Oscillibacter;s__uncultured_organism							0.0046	NT						
d__Bacteria;p__Firmicutes;c__Clostridia;o__Oscillospirales;f__Oscillospiraceae;g__UCG-003;__	0.02 (0.17)	0.06 (0.12)	0 (0.07)	0.1 (0.17)	0 (0.06)	0 (0)	0.005	NT						
d__Bacteria;p__Firmicutes;c__Clostridia;o__Oscillospirales;f__Oscillospiraceae;g__UCG-005;__	0 (0.03)	0 (0.02)	0 (0.03)	0 (0)	0 (0)	0 (0)	0.041	NT						
d__Bacteria;p__Firmicutes;c__Clostridia;o__Oscillospirales;f__Oscillospiraceae;g__UCG-005;s__unidentified	0 (0)	0 (0)	0 (0)	0.08 (0.15)	0.08 (0.18)	0 (0)	**< .0001**	**0.034**	**0.034**	1	1	1	0.9999	**0.034**
d__Bacteria;p__Firmicutes;c__Clostridia;o__Oscillospirales;f__Oscillospiraceae;g__uncultured;__	0.24 (0.37)	0.11 (0.12)	0.12 (0.27)	0.13 (0.24)	0 (0)	0 (0)	**< .0001**	0.5851	0.0944	0.0802	0.166	0.2227	0.0804	**0.0276**

d__Bacteria;p__Firmicutes;c__Clostridia;o__Oscillospirales;f__Oscillospiraceae;g__uncultured;s__Clostridiales_bacterium	0.52 (1.4)	0.5 (0.94)	0.55 (0.88)	1.08 (1.49)	1.04 (1.45)	0.28 (0.35)	0.0028	NT						
d__Bacteria;p__Firmicutes;c__Clostridia;o__Oscillospirales;f__Ruminococcaceae;__;__	1.14 (0.56)	0.68 (0.38)	0.28 (0.19)	0.25 (0.5)	0.09 (0.13)	0.02 (0.06)	**< .0001**	**0.035**	**0.0011**	**0.0111**	0.1787	**0.0017**	**0.0141**	**0.0017**
d__Bacteria;p__Firmicutes;c__Clostridia;o__Oscillospirales;f__Ruminococcaceae;g__Anaerotruncus;s__Anaerotruncus_colihominis	0.1 (0.14)	0.06 (0.03)	0.05 (0.05)	0.01 (0.04)	0 (0.02)	0 (0.03)	**< .0001**	**0.0071**	0.1022	0.1792	**0.0489**	**0.0347**	0.9768	0.9861
d__Bacteria;p__Firmicutes;c__Clostridia;o__Oscillospirales;f__Ruminococcaceae;g__Anaerotruncus;s__Anaerotruncus_sp.	0.07 (0.05)	0 (0)	0 (0.01)	0 (0.03)	0 (0.01)	0 (0)	**< .0001**	**0.0262**	0.8946	0.4934	**0.0002**	**0.0018**	0.9295	0.1567
d__Bacteria;p__Firmicutes;c__Clostridia;o__Oscillospirales;f__Ruminococcaceae;g__Incertae_Sedis;__	1.04 (1.07)	0.19 (0.39)	0.16 (0.22)	0.33 (0.62)	0.07 (0.37)	0.02 (0.04)	**< .0001**	0.1049	0.7529	0.0555	**0.0022**	**0.0009**	0.1575	**0.0017**
d__Bacteria;p__Firmicutes;c__Clostridia;o__Oscillospirales;f__Ruminococcaceae;g__Incertae_Sedis;s__uncultured_Clostridiales	0.11 (0.18)	0 (0.1)	0 (0)	0.04 (0.16)	0.03 (0.1)	0.06 (0.08)	0.0029	NT						
d__Bacteria;p__Firmicutes;c__Clostridia;o__Oscillospirales;f__Ruminococcaceae;g__Negativibacillus;s__unidentified	0.13 (0.2)	0 (0.06)	0 (0.06)	0 (0)	0 (0)	0 (0)	**< .0001**	**0.0003**	0.5871	0.4517	**0.0011**	**0.0009**	1	0.9976
d__Bacteria;p__Firmicutes;c__Clostridia;o__Oscillospirales;f__Ruminococcaceae;g__UBA1819;__	0 (0.02)	0 (0)	0 (0)	0.02 (0.06)	0.03 (0.05)	0 (0.02)	0.0015	NT						
d__Bacteria;p__Firmicutes;c__Clostridia;o__Oscillospirales;f__Ruminococcaceae;g__uncultured;s__human_gut	0.22 (0.23)	0.27 (0.35)	0.3 (0.28)	0.03 (0.09)	0.05 (0.07)	0.02 (0.05)	**< .0001**	**0.0244**	0.12	**0.0005**	0.9325	0.2271	0.9233	0.9358
d__Bacteria;p__Firmicutes;c__Clostridia;o__Oscillospirales;f__Ruminococcaceae;g__uncultured;s__uncultured_Clostridium	0.03 (0.06)	0.01 (0.07)	0.04 (0.07)	0 (0)	0 (0)	0 (0)	**< .0001**	**0.0186**	0.5414	**0.0051**	0.9995	0.9993	1	0.7365
d__Bacteria;p__Firmicutes;c__Clostridia;o__Oscillospirales;f__UCG-010;g__UCG-010;__	0.01 (0.05)	0 (0.05)	0 (0.01)	0.03 (0.1)	0 (0)	0 (0.01)	0.0148	NT						
d__Bacteria;p__Firmicutes;c__Clostridia;o__Oscillospirales;f__UCG-010;g__UCG-010;s__uncultured_organism	0.35 (0.45)	0 (0.44)	0 (0)	0 (0.13)	0 (0.1)	0 (0)	**< .0001**	**0.0014**	0.9476	0.9424	0.1903	**0.0003**	0.9963	0.1567
d__Bacteria;p__Firmicutes;c__Clostridia;o__Peptococcales;f__Peptococcaceae;g__uncultured;__	0.12 (0.13)	0.19 (0.14)	0.12 (0.14)	0 (0.03)	0 (0.06)	0 (0.04)	**< .0001**	**0.0104**	**0.0071**	**0.0011**	0.5682	1	0.9985	0.9833
d__Bacteria;p__Firmicutes;c__Clostridia;o__Peptococcales;f__Peptococcaceae;g__uncultured;s__unidentified	6.21 (2.85)	2.36 (3.94)	1.43 (2.85)	3.37 (3.05)	1.9 (3.2)	0.37 (1.99)	**< .0001**	**0.0414**	0.9794	0.9606	0.0908	**0.0014**	0.8708	0.1568
d__Bacteria;p__Firmicutes;c__Clostridia;o__Peptostreptococcales-Tissierellales;__;__;__	1.38 (1.74)	0.61 (0.85)	0.19 (0.51)	0.01 (0.17)	0 (0)	0 (0)	**< .0001**	**0.0007**	**0.0001**	**0.0052**	0.4913	**0.0413**	0.0763	0.4071
d__Bacteria;p__Firmicutes;c__Clostridia;o__Peptostreptococcales-Tissierellales;f__Anaerovoracaceae;g__[Eubacterium]_brachy_group;s__uncultured_organism	0.05 (0.07)	0.03 (0.03)	0.03 (0.04)	0.08 (0.18)	0.02 (0.07)	0.01 (0.02)	0.0167	NT						
d__Bacteria;p__Firmicutes;c__Clostridia;o__Peptostreptococcales-Tissierellales;f__Anaerovoracaceae;g__Family_XIII_AD3011_group;s__gut_metagenome	0.17 (0.17)	0.05 (0.13)	0 (0.07)	0 (0)	0 (0)	0 (0)	**< .0001**	**0.0005**	**0.0005**	0.1567	0.4721	**0.0385**	1	1
d__Bacteria;p__Firmicutes;c__Clostridia;o__Peptostreptococcales-Tissierellales;f__Peptostreptococcaceae;__;__	3.2 (1.73)	0.95 (0.98)	0.81 (2.27)	0 (0)	0 (0)	0 (0)	**< .0001**	**0.0001**	**0.0005**	**0.0138**	**0.0078**	**0.048**	1	1
d__Bacteria;p__Firmicutes;c__Clostridia;o__Peptostreptococcales-Tissierellales;f__Peptostreptococcaceae;g__uncultured;__	3.3 (1.64)	0.94 (1.07)	0.87 (2.43)	0 (0)	0 (0)	0 (0)	**< .0001**	**0.0001**	**0.0009**	**0.0138**	**0.0141**	**0.0407**	0.9424	1
d__Bacteria;p__Proteobacteria;c__Gammaproteobacteria;o__Enterobacterales;__;__;__	0.21 (0.55)	0.38 (1.33)	0.34 (0.97)	0.05 (0.32)	0.03 (0.09)	0 (0.03)	0.0007	NT						
d__Bacteria;p__Verrucomicrobiota;c__Verrucomicrobiae;o__Verrucomicrobiales;f__Akkermansiaceae;g__Akkermansia;s__Akkermansia_muciniphila	0 (0.01)	0 (0.01)	0 (0.01)	17.32 (19.9)	0 (0)	21.17 (18.52)	**< .0001**	**0.0318**	0.953	**0.0068**	1	1	**0.0226**	0.6813

All data (n = 12 per group) are expressed as the median and interquartile range (IQR). Only species that are detected in more than 10 % samples were selected and processed for comparizons among the groups. When a p value was less than 0.00049 in the KW test comparing the six groups, the Steel-Dwass test was applied to evaluate differences in the relative abundance between the indicated two groups.The p value of 0.00049 in the KW test was calculated using the Bonferroni correction based on the total number of tests (102, p = 0.05/102 = 0.00049). Bold figures indicate a significant difference between the indicated two groups ($p < 0.05$). G1, G2, and G3 show the 1st, 2nd, and 3rd generation, respectively. KW, Kruskal-Wallis test; CON, a group fed control diets without KES; KES, a group fed 1-kestose-supplemented diets; NT, not tested.
