# Supplementary material for: Dietary supplementation with 1‐kestose induces altered locomotor activity and increased striatal dopamine levels with a change in gut microbiota in male mice
Source: Physiol Rep. 2023 Dec 6;11(23):e15882. doi: 10.14814/phy2.15882 (PMC10698829; doi:10.14814/phy2.15882)
Supplement: Supplementary file 4 — Table S4. [file PHY2-11-e15882-s001.pdf]

Supplementary Table 4. Correlations between striatal dopamine levels and bacterial genera in CON and KES groups

	CON		KES	
	Spearman ρ value	p value	Spearman ρ value	p value
d__Bacteria;p__Actinobacteriota;c__Actinobacteria;o__Bifidobacteriales;f__Bifidobacteriaceae;g__Bifidobacterium	0.27849	0.10003	0.40310	0.01809
d__Bacteria;p__Actinobacteriota;c__Coriobacteriia;o__Coriobacteriales;__;__	0.40711	0.01373	-0.41388	0.01496
d__Bacteria;p__Actinobacteriota;c__Coriobacteriia;o__Coriobacteriales;f__Eggerthellaceae;__	0.10682	0.53520	-0.16119	0.36244
d__Bacteria;p__Bacteroidota;c__Bacteroidia;o__Bacteroidales;f__Bacteroidaceae;g__Bacteroides	-0.32059	0.05661	0.27508	0.11537
d__Bacteria;p__Bacteroidota;c__Bacteroidia;o__Bacteroidales;f__Muribaculaceae;g__Muribaculaceae	0.30231	0.07312	-0.17403	0.32496
d__Bacteria;p__Bacteroidota;c__Bacteroidia;o__Bacteroidales;f__Tannerellaceae;__	-0.18198	0.28812	-0.21772	0.21610
d__Bacteria;p__Deferribacterota;c__Deferribacteres;o__Deferribacterales;f__Deferribacteraceae;g__Mucispirillum	-0.21839	0.20069	-0.44162	0.00893
d__Bacteria;p__Firmicutes;c__Bacilli;o__Erysipelotrichales;f__Erysipelatoclostridiaceae;g__Candidatus_Stoquefichus	-0.26550	0.11758	-0.22246	0.20603
d__Bacteria;p__Firmicutes;c__Bacilli;o__Erysipelotrichales;f__Erysipelatoclostridiaceae;g__Erysipelatoclostridium	0.19537	0.25351	-0.33537	0.05251
d__Bacteria;p__Firmicutes;c__Bacilli;o__Erysipelotrichales;f__Erysipelotrichaceae;__	-0.37867	0.02276	-0.31289	0.07158
d__Bacteria;p__Firmicutes;c__Bacilli;o__Erysipelotrichales;f__Erysipelotrichaceae;g__Erysipelotrichaceae	-0.13064	0.44757	-0.41859	0.01375
d__Bacteria;p__Firmicutes;c__Bacilli;o__Erysipelotrichales;f__Erysipelotrichaceae;g__Faecalibaculum	0.29634	0.07927	0.44414	0.00850
d__Bacteria;p__Firmicutes;c__Bacilli;o__Erysipelotrichales;f__Erysipelotrichaceae;g__Turicibacter	-0.21361	0.21096	-0.53418	0.00114
d__Bacteria;p__Firmicutes;c__Bacilli;o__Erysipelotrichales;f__Erysipelotrichaceae;g__uncultured	-0.26848	0.11337	-0.23120	0.18832
d__Bacteria;p__Firmicutes;c__Bacilli;o__Lactobacillales;f__Enterococcaceae;g__Enterococcus	-0.34677	0.03827	-0.61127	**0.00012**
d__Bacteria;p__Firmicutes;c__Bacilli;o__Lactobacillales;f__Lactobacillaceae;g__Lactobacillus	-0.13533	0.43130	-0.43842	0.00950
d__Bacteria;p__Firmicutes;c__Bacilli;o__Lactobacillales;f__Streptococcaceae;g__Lactococcus	-0.18121	0.29020	-0.41329	0.01512
d__Bacteria;p__Firmicutes;c__Bacilli;o__Lactobacillales;f__Streptococcaceae;g__Streptococcus	0.28482	0.09224	0.05545	0.75543
d__Bacteria;p__Firmicutes;c__Bacilli;o__RF39;f__RF39;g__RF39	-0.17583	0.30500	-0.46302	0.00582
d__Bacteria;p__Firmicutes;c__Bacilli;o__Staphylococcales;f__Staphylococcaceae;g__Staphylococcus	-0.40470	0.01435	-0.56823	**0.00046**
d__Bacteria;p__Firmicutes;c__Clostridia;__;__;__	-0.09524	0.58059	-0.45811	0.00644
d__Bacteria;p__Firmicutes;c__Clostridia;o__Christensenellales;f__Christensenellaceae;__	0.02879	0.86760	-0.34713	0.04428
d__Bacteria;p__Firmicutes;c__Clostridia;o__Christensenellales;f__Christensenellaceae;g__Christensenellaceae_R-7_group	0.15707	0.36028	-0.33566	0.05229
d__Bacteria;p__Firmicutes;c__Clostridia;o__Clostridia_UCG-014;f__Clostridia_UCG-014;g__Clostridia_UCG-014	-0.19379	0.25744	-0.51177	0.00198
d__Bacteria;p__Firmicutes;c__Clostridia;o__Clostridia_vadinBB60_group;f__Clostridia_vadinBB60_group;g__Clostridia_vadinBB60_group	-0.41066	0.01285	-0.35163	0.04142
d__Bacteria;p__Firmicutes;c__Clostridia;o__Clostridiales;f__Clostridiaceae;__	-0.18107	0.29057	-0.20906	0.23540
d__Bacteria;p__Firmicutes;c__Clostridia;o__Clostridiales;f__Clostridiaceae;g__Candidatus_Arthromitus	-0.20259	0.23603	0.10082	0.57049
d__Bacteria;p__Firmicutes;c__Clostridia;o__Lachnospirales;__;__	-0.25531	0.13288	-0.17376	0.32572
d__Bacteria;p__Firmicutes;c__Clostridia;o__Lachnospirales;f__Lachnospiraceae;__	-0.19254	0.26059	-0.21558	0.22076
d__Bacteria;p__Firmicutes;c__Clostridia;o__Lachnospirales;f__Lachnospiraceae;g__[Acetivibrio]_ethanolgignens_group	-0.17801	0.29896	NA	
d__Bacteria;p__Firmicutes;c__Clostridia;o__Lachnospirales;f__Lachnospiraceae;g__A2	-0.18816	0.27178	-0.28067	0.10786
d__Bacteria;p__Firmicutes;c__Clostridia;o__Lachnospirales;f__Lachnospiraceae;g__Acetatifactor	-0.28932	0.08700	-0.33659	0.05160
d__Bacteria;p__Firmicutes;c__Clostridia;o__Lachnospirales;f__Lachnospiraceae;g__Blautia	0.10837	0.52928	0.13277	0.45412
d__Bacteria;p__Firmicutes;c__Clostridia;o__Lachnospirales;f__Lachnospiraceae;g__Dorea	0.06837	0.69196	-0.30598	0.07842
d__Bacteria;p__Firmicutes;c__Clostridia;o__Lachnospirales;f__Lachnospiraceae;g__GCA-900066575	-0.18147	0.28951	-0.47288	0.00474
d__Bacteria;p__Firmicutes;c__Clostridia;o__Lachnospirales;f__Lachnospiraceae;g__Lachnoclostridium	-0.26615	0.11665	-0.35493	0.03942
d__Bacteria;p__Firmicutes;c__Clostridia;o__Lachnospirales;f__Lachnospiraceae;g__Lachnospiraceae_FCS020_group	0.27544	0.10397	0.03219	0.85659
d__Bacteria;p__Firmicutes;c__Clostridia;o__Lachnospirales;f__Lachnospiraceae;g__Lachnospiraceae_UCG-004	0.37246	0.02528	-0.09274	0.60190
d__Bacteria;p__Firmicutes;c__Clostridia;o__Lachnospirales;f__Lachnospiraceae;g__Lachnospiraceae_UCG-006	-0.11274	0.51268	-0.21528	0.22143
d__Bacteria;p__Firmicutes;c__Clostridia;o__Lachnospirales;f__Lachnospiraceae;g__Marvinbryantia	-0.26898	0.11266	-0.31979	0.06523
d__Bacteria;p__Firmicutes;c__Clostridia;o__Lachnospirales;f__Lachnospiraceae;g__Roseburia	-0.29298	0.08290	-0.29480	0.09055
d__Bacteria;p__Firmicutes;c__Clostridia;o__Lachnospirales;f__Lachnospiraceae;g__Sellimonas	0.35932	0.03138	-0.06170	0.72884
d__Bacteria;p__Firmicutes;c__Clostridia;o__Lachnospirales;f__Lachnospiraceae;g__Tuzzerella	-0.00695	0.96791	-0.25264	0.14943
d__Bacteria;p__Firmicutes;c__Clostridia;o__Monoglobales;f__Monoglobaceae;g__Monoglobus	0.01985	0.90854	-0.22362	0.20361
d__Bacteria;p__Firmicutes;c__Clostridia;o__Oscillospirales;__;__	-0.25766	0.12923	-0.40675	0.01697
d__Bacteria;p__Firmicutes;c__Clostridia;o__Oscillospirales;f__[Eubacterium]_coprostanoligenes_group;g__[Eubacterium]_coprostanoligenes_group	-0.40160	0.01519	-0.19939	0.25825
d__Bacteria;p__Firmicutes;c__Clostridia;o__Oscillospirales;f__Oscillospiraceae;__	-0.41776	0.01124	-0.40932	0.01622
d__Bacteria;p__Firmicutes;c__Clostridia;o__Oscillospirales;f__Oscillospiraceae;g__Colidextribacter	-0.26512	0.11812	-0.57861	**0.00034**
d__Bacteria;p__Firmicutes;c__Clostridia;o__Oscillospirales;f__Oscillospiraceae;g__Intestinimonas	-0.15921	0.35367	-0.48122	0.00396
d__Bacteria;p__Firmicutes;c__Clostridia;o__Oscillospirales;f__Oscillospiraceae;g__NK4A214_group	-0.09834	0.56826	-0.50610	0.00226
d__Bacteria;p__Firmicutes;c__Clostridia;o__Oscillospirales;f__Oscillospiraceae;g__Oscillibacter	-0.25508	0.13324	-0.39740	0.01995
d__Bacteria;p__Firmicutes;c__Clostridia;o__Oscillospirales;f__Oscillospiraceae;g__Oscillospira	0.02379	0.89047	-0.39771	0.01984
d__Bacteria;p__Firmicutes;c__Clostridia;o__Oscillospirales;f__Oscillospiraceae;g__UCG-003	-0.13006	0.44964	-0.62578	**0.00008**
d__Bacteria;p__Firmicutes;c__Clostridia;o__Oscillospirales;f__Oscillospiraceae;g__UCG-005	-0.10192	0.55418	-0.35654	0.03847
d__Bacteria;p__Firmicutes;c__Clostridia;o__Oscillospirales;f__Oscillospiraceae;g__uncultured	-0.32209	0.05540	-0.48602	0.00357
d__Bacteria;p__Firmicutes;c__Clostridia;o__Oscillospirales;f__Ruminococcaceae;__	-0.42214	0.01033	-0.50673	0.00223
d__Bacteria;p__Firmicutes;c__Clostridia;o__Oscillospirales;f__Ruminococcaceae;g__Anaerotruncus	-0.30536	0.07014	-0.40059	0.01889
d__Bacteria;p__Firmicutes;c__Clostridia;o__Oscillospirales;f__Ruminococcaceae;g__Incertae_Sedis	-0.41441	0.01198	-0.21161	0.22959
d__Bacteria;p__Firmicutes;c__Clostridia;o__Oscillospirales;f__Ruminococcaceae;g__Negativibacillus	-0.25321	0.13620	-0.07464	0.67485
d__Bacteria;p__Firmicutes;c__Clostridia;o__Oscillospirales;f__Ruminococcaceae;g__UBA1819	0.01784	0.91774	-0.26072	0.13642
d__Bacteria;p__Firmicutes;c__Clostridia;o__Oscillospirales;f__Ruminococcaceae;g__uncultured	-0.02149	0.90098	-0.38629	0.02403
d__Bacteria;p__Firmicutes;c__Clostridia;o__Oscillospirales;f__UCG-010;g__UCG-010	-0.31126	0.06462	-0.46788	0.00527
d__Bacteria;p__Firmicutes;c__Clostridia;o__Peptococcales;f__Peptococcaceae;g__uncultured	-0.24916	0.14280	-0.35473	0.03954
d__Bacteria;p__Firmicutes;c__Clostridia;o__Peptostreptococcales-Tissierellales;__;__	-0.15303	0.37289	-0.20442	0.24619
d__Bacteria;p__Firmicutes;c__Clostridia;o__Peptostreptococcales-Tissierellales;f__Anaerovoracaceae;g__[Eubacterium]_brachy_group	0.01819	0.91615	-0.27345	0.11763
d__Bacteria;p__Firmicutes;c__Clostridia;o__Peptostreptococcales-Tissierellales;f__Anaerovoracaceae;g__Family_XIII_AD3011_group	-0.25708	0.13013	NA	
d__Bacteria;p__Firmicutes;c__Clostridia;o__Peptostreptococcales-Tissierellales;f__Peptostreptococcaceae;__	-0.31546	0.06091	NA	
d__Bacteria;p__Firmicutes;c__Clostridia;o__Peptostreptococcales-Tissierellales;f__Peptostreptococcaceae;g__uncultured	-0.32938	0.04980	NA	
d__Bacteria;p__Proteobacteria;c__Gammaproteobacteria;o__Enterobacterales;__;__	-0.04250	0.80560	-0.43161	0.01081
d__Bacteria;p__Verrucomicrobiota;c__Verrucomicrobiae;o__Verrucomicrobiales;f__Akkermansiaceae;g__Akkermansia	0.03553	0.83701	0.28561	0.10155

Correlations between striatal dopamine levels and relative abundance at bacterial genera were evaluated using Spearman's rank-order test. A p value less than 0.000714 was considered to be significant. This p value was calculated using the Bonferroni correction based on the total number of tests (70, p = 0.05/70 = 0.000714). Bold figures indicate a significant difference between the two indicated variables. CON, a group fed control diets; KES, a group fed kestose-supplemented diets; NA, not applicable.
