# Supplementary material for: Dietary supplementation with 1‐kestose induces altered locomotor activity and increased striatal dopamine levels with a change in gut microbiota in male mice
Source: Physiol Rep. 2023 Dec 6;11(23):e15882. doi: 10.14814/phy2.15882 (PMC10698829; doi:10.14814/phy2.15882)
Supplement: Supplementary file 5 — Data S1. [file PHY2-11-e15882-s003.docx]

**Supplemental Materials available at**

**URL:** <https://figshare.com/search?q=10.6084%2Fm9.figshare.23691387>

**DOI**: [https://doi.org/10.6084/m9.figshare.23691387](http://dx.doi.org/10.6084/m9.figshare.23691387)
